# Supplementary material for: RBM38 Reverses Sorafenib Resistance in Hepatocellular Carcinoma Cells by Combining and Promoting lncRNA-GAS5
Source: Cancers (Basel). 2023 May 24;15(11):2897. doi: 10.3390/cancers15112897 (PMC10252096; doi:10.3390/cancers15112897)

Figure 1

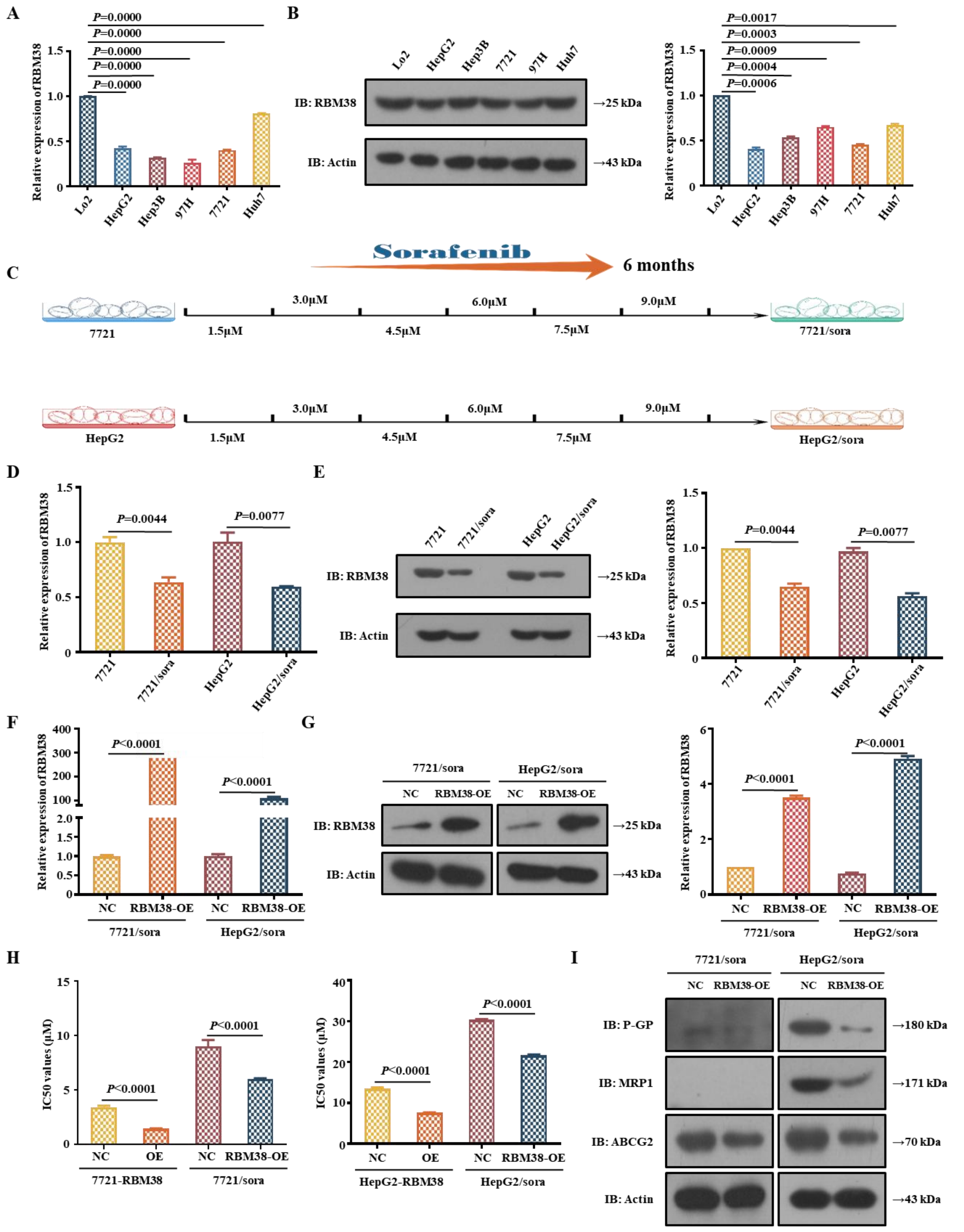
A
B
C
D
E
F
G
H
I
Sorafenib
6 months
7721
7721/sora
HepG2
HepG2/sora
1.5μM
3.0μM
4.5μM
6.0μM
7.5μM
9.0μM
IB: RBM38
IB: Actin
IB: P-GP
IB: MRP1
IB: ABCG2
→25 kDa
→43 kDa
→180 kDa
→171 kDa
→70 kDa
Relative expression of RBM38
IC50 values (μM)
NC
RBM38-OE
OE
7721-RBM38
HepG2-RBM38
Lo2
Hep3B
97H
Huh7

Figure 1A. Western blot analysis of the expression and protein levels of RBM38 in HCC and normal liver cell lines.

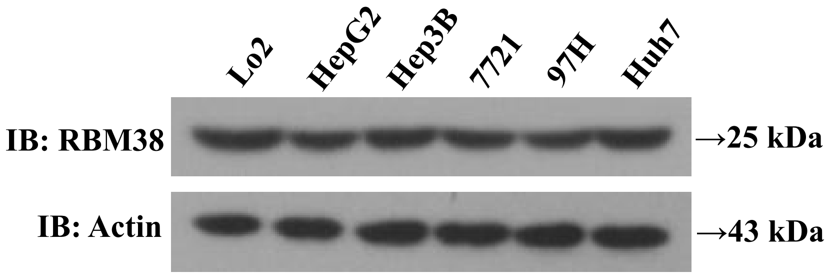

Figure 1A. Western blot analysis of the expression and protein levels of RBM38 in HCC and normal liver cell lines.

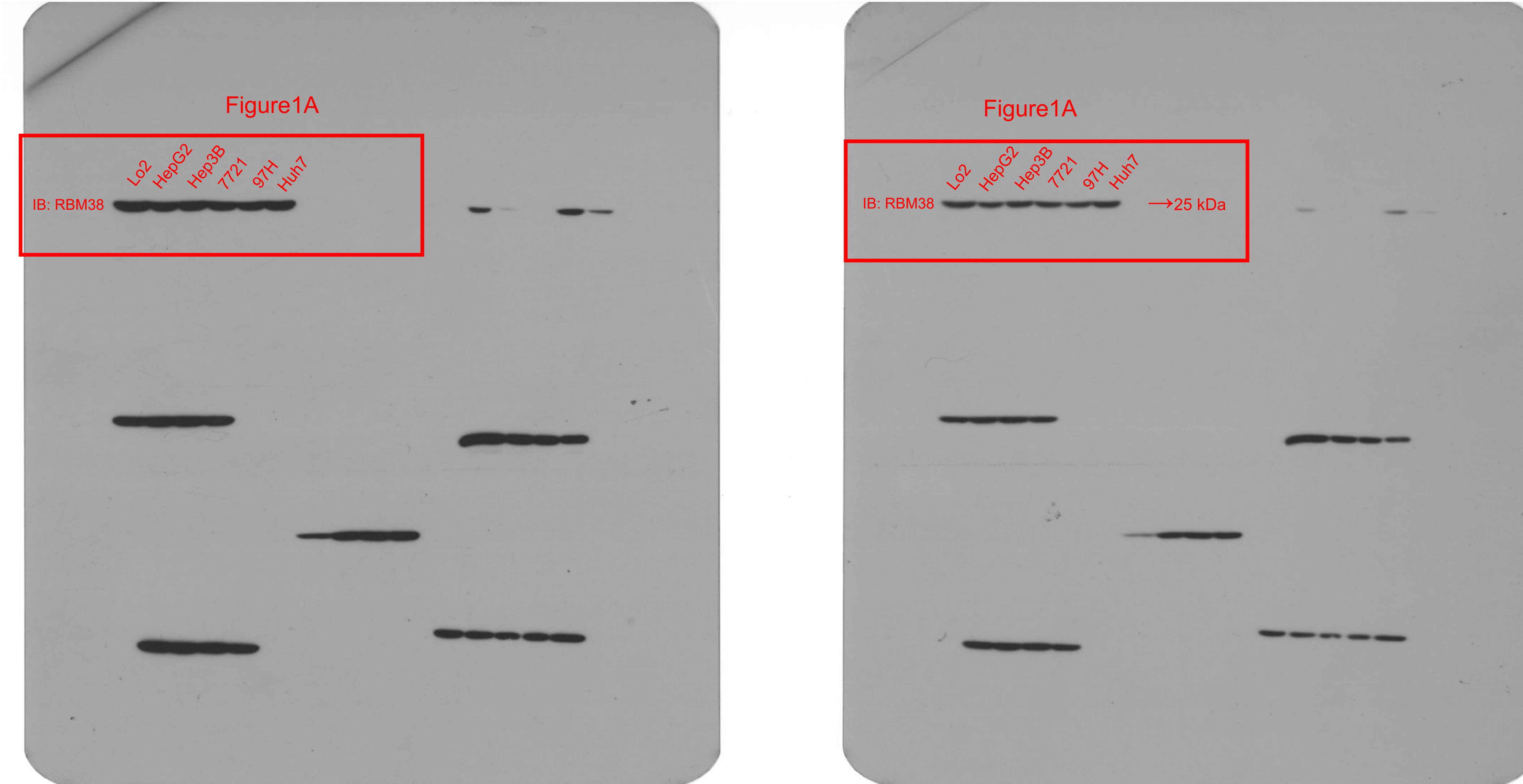

Figure 1A. Western blot analysis of the expression and protein levels of RBM38 in HCC and normal liver cell lines.

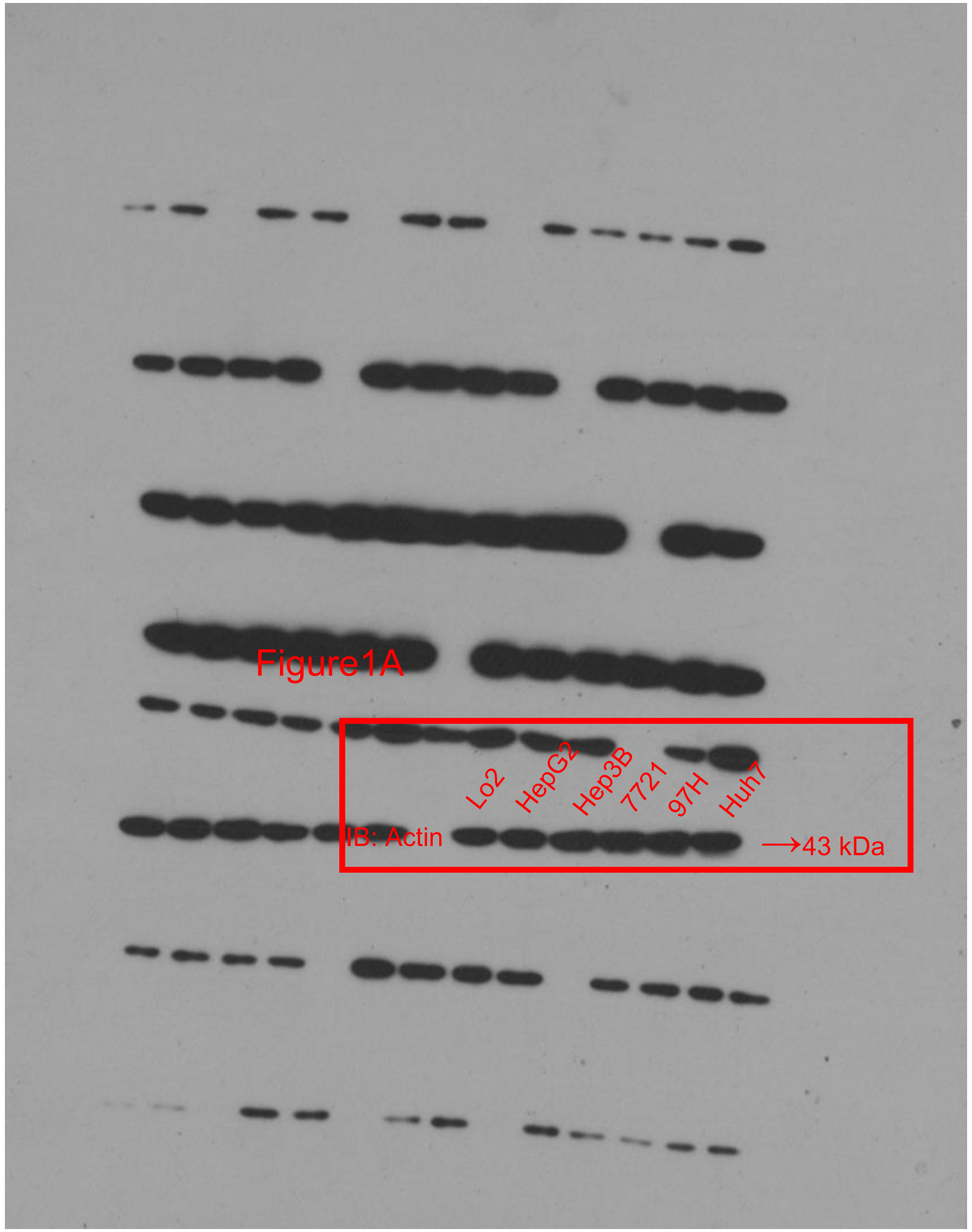

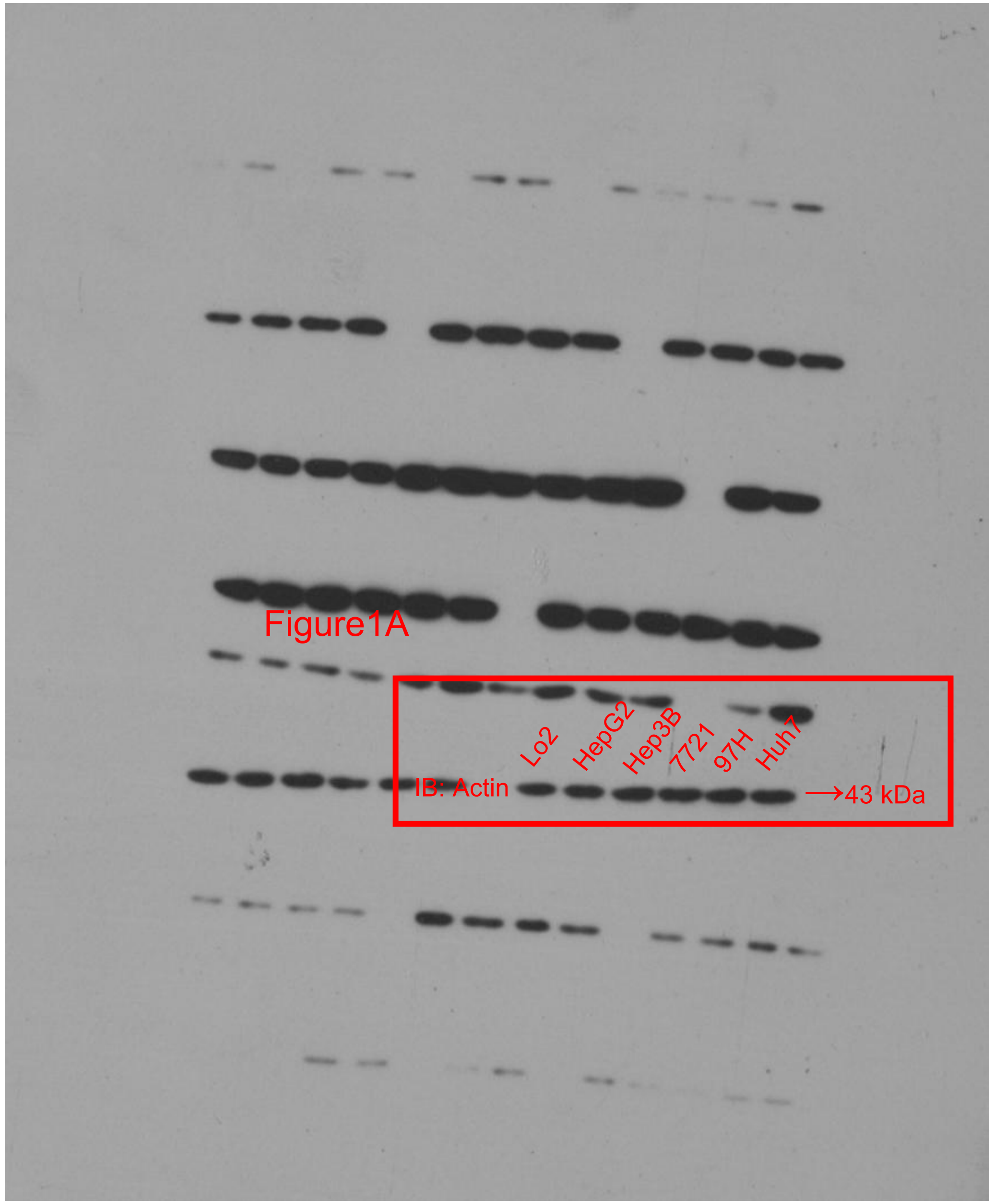

Figure 1C. Western blot analysis of the expression and protein levels of RBM38 in PCL and drug-resistant HCC cell line.

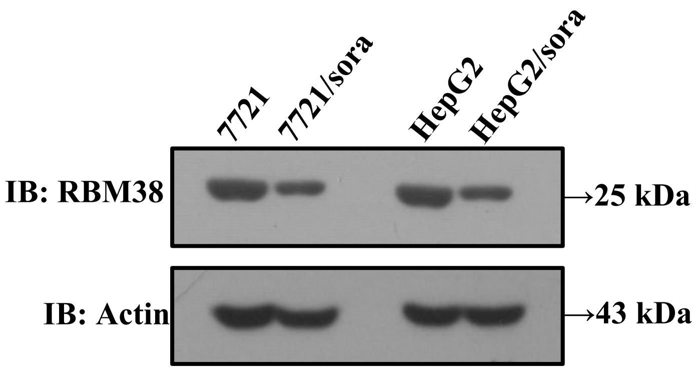

Figure 1C. Western blot analysis of the expression and protein levels of RBM38 in PCL and drug-resistant HCC cell line.

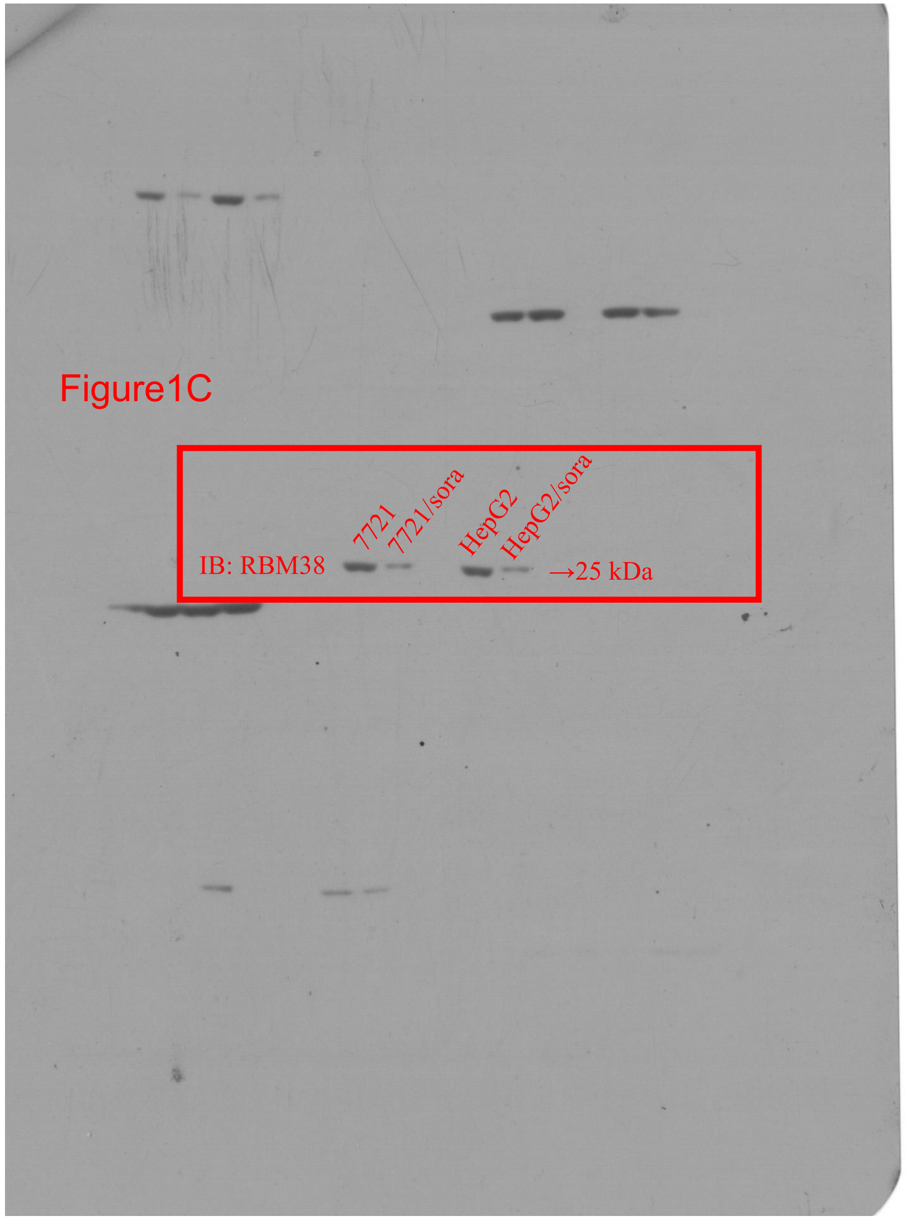

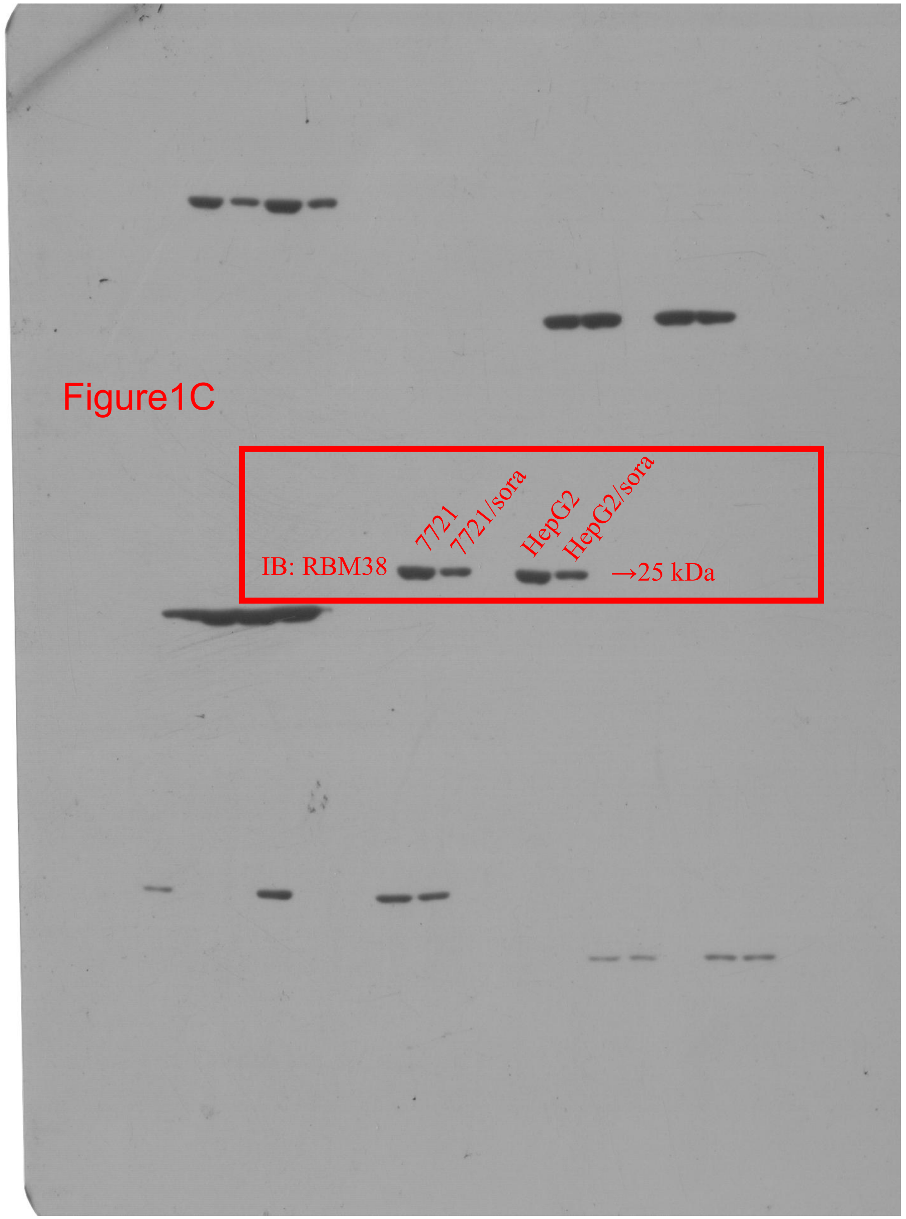

Figure 1C. Western blot analysis of the expression and protein levels of RBM38 in PCL and drug-resistant HCC cell line.

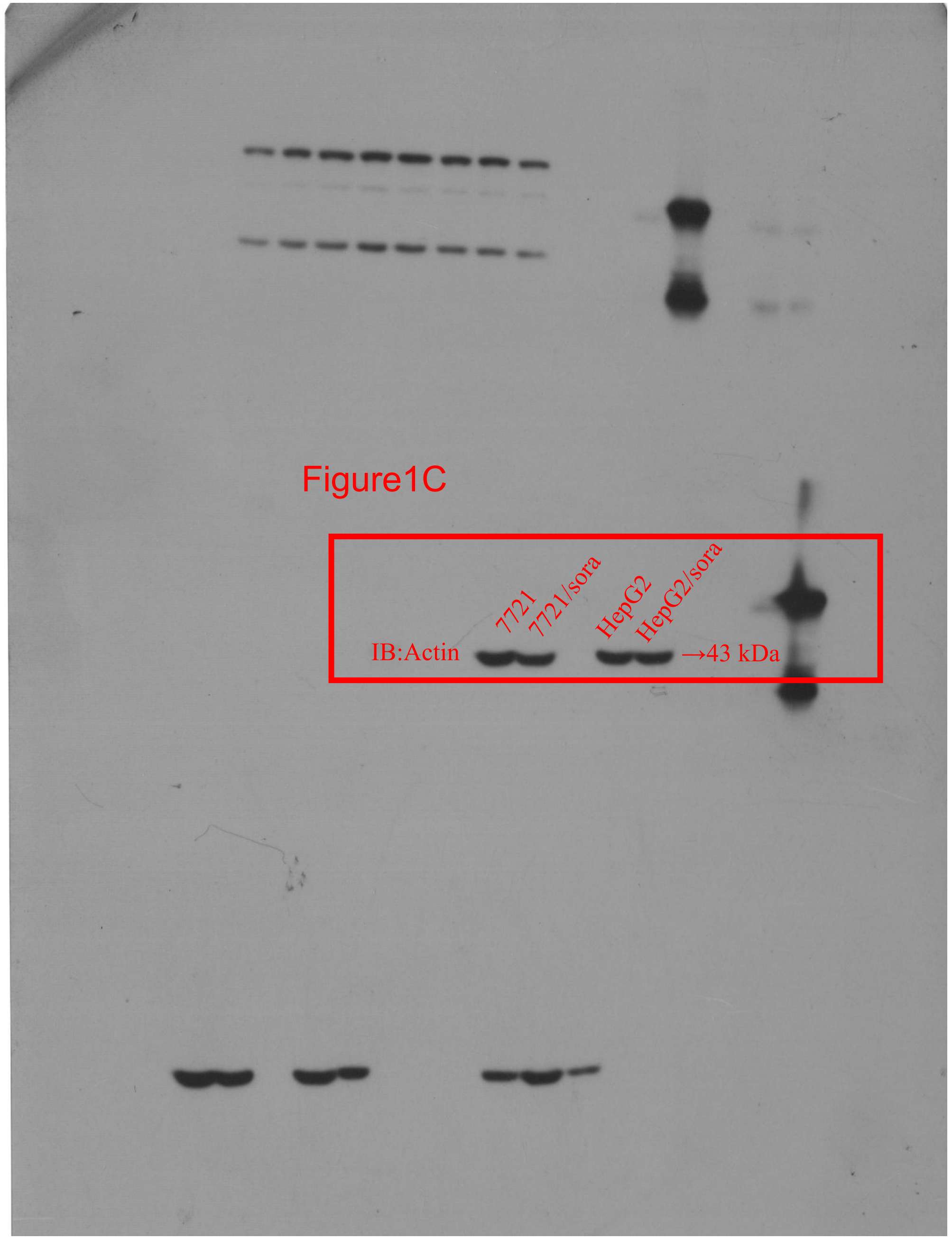

Figure 1D. Overexpression (OE) efficiency of RBM38 in PCLs and drug-resistant HCC cells based on western blot analysis, compared with the negative control (NC).

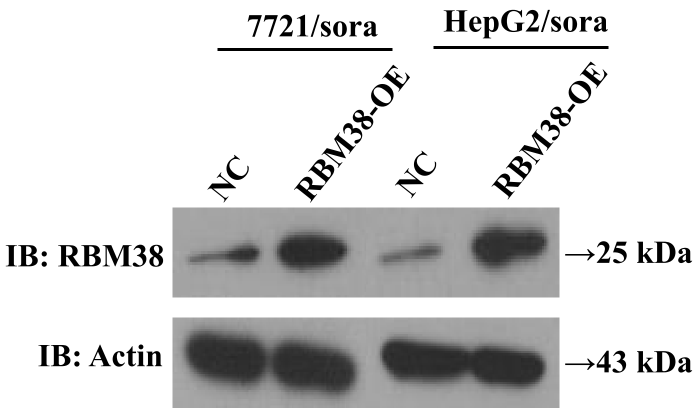

Figure 1D. Overexpression (OE) efficiency of RBM38 in PCLs and drug-resistant HCC cells based on western blot analysis, compared with the negative control (NC).

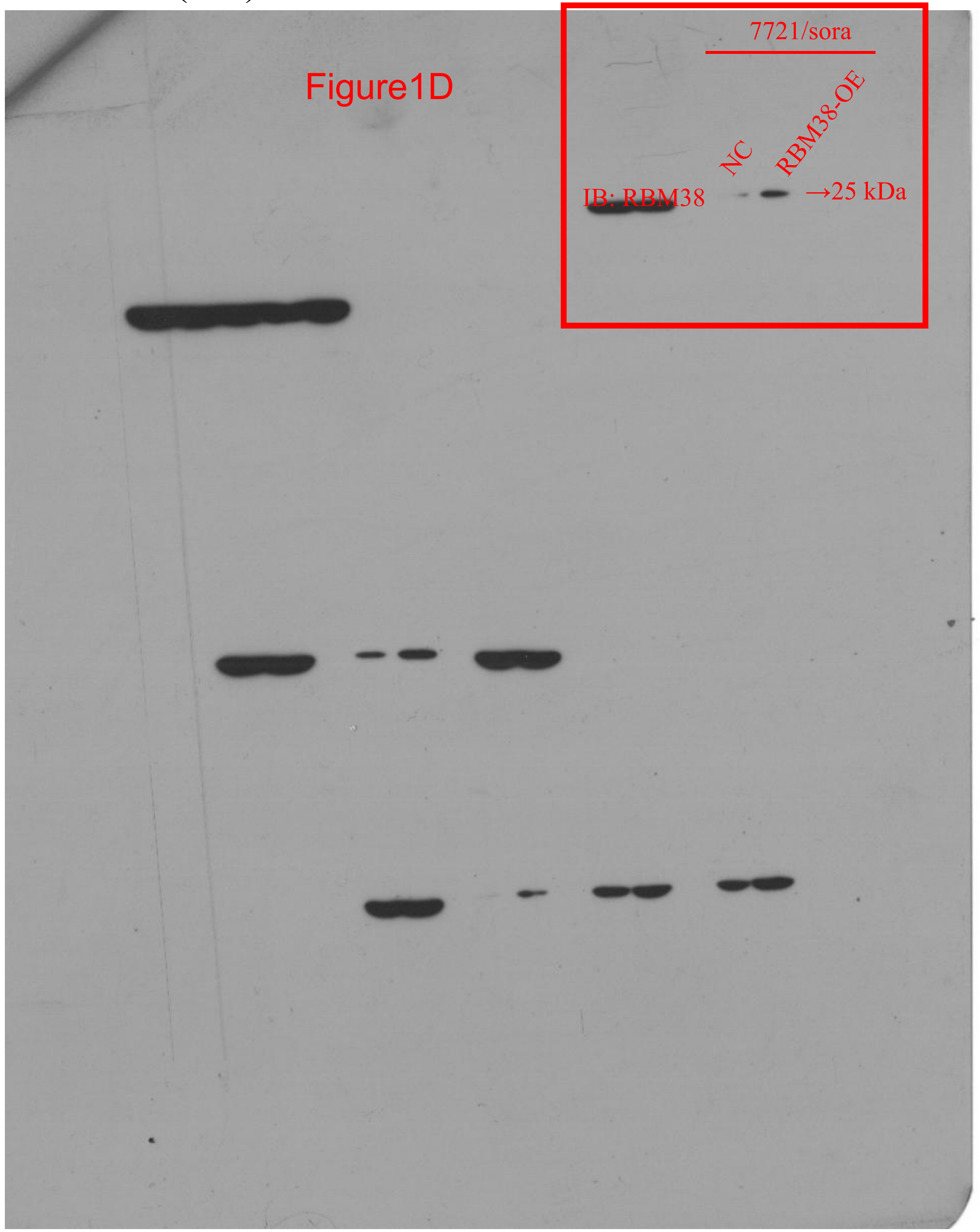

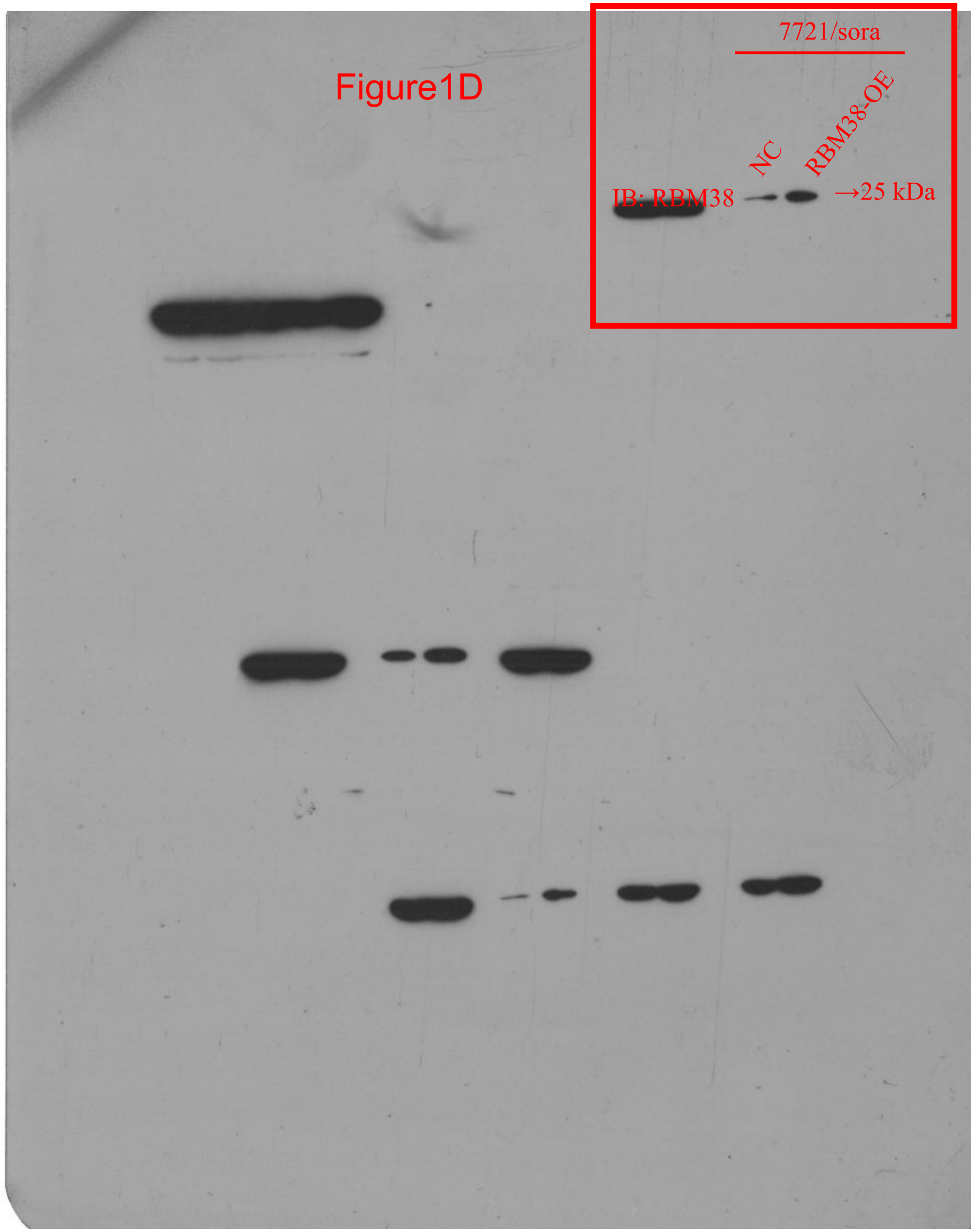

Figure 1D. Overexpression (OE) efficiency of RBM38 in PCLs and drug-resistant HCC cells based on western blot analysis, compared with the negative control (NC).

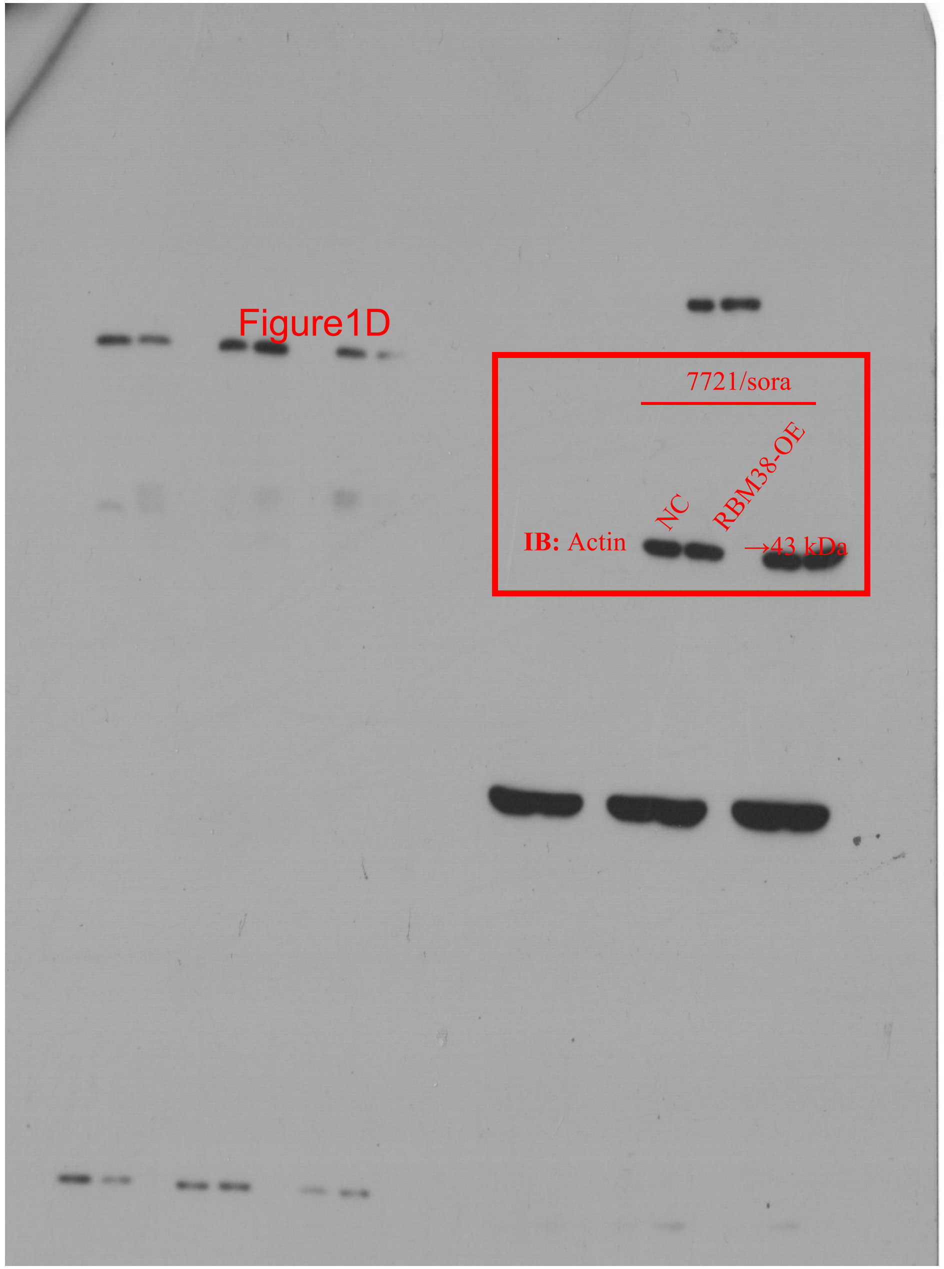

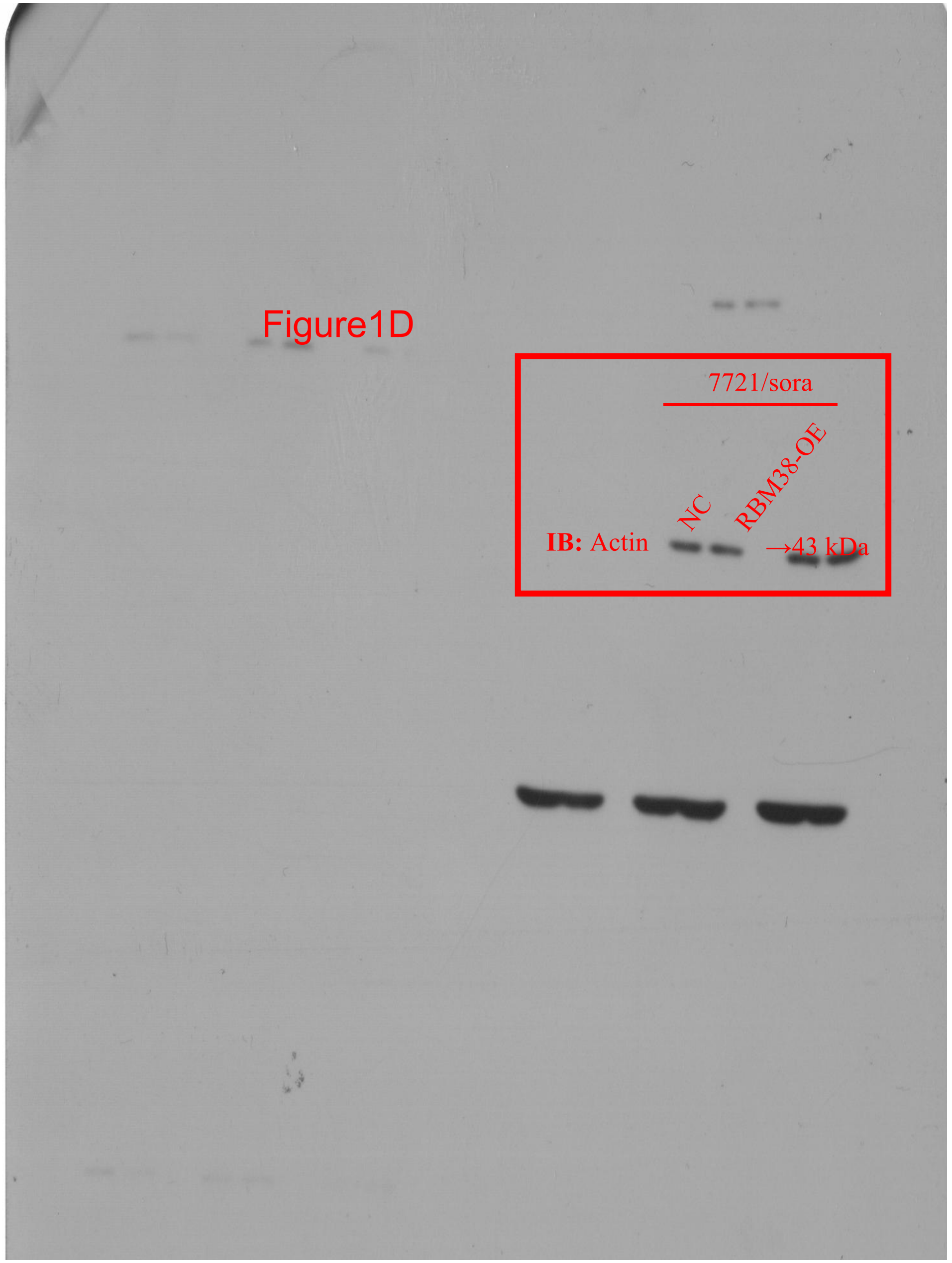

Figure 1D. Overexpression (OE) efficiency of RBM38 in PCLs and drug-resistant HCC cells based on western blot analysis, compared with the negative control (NC).

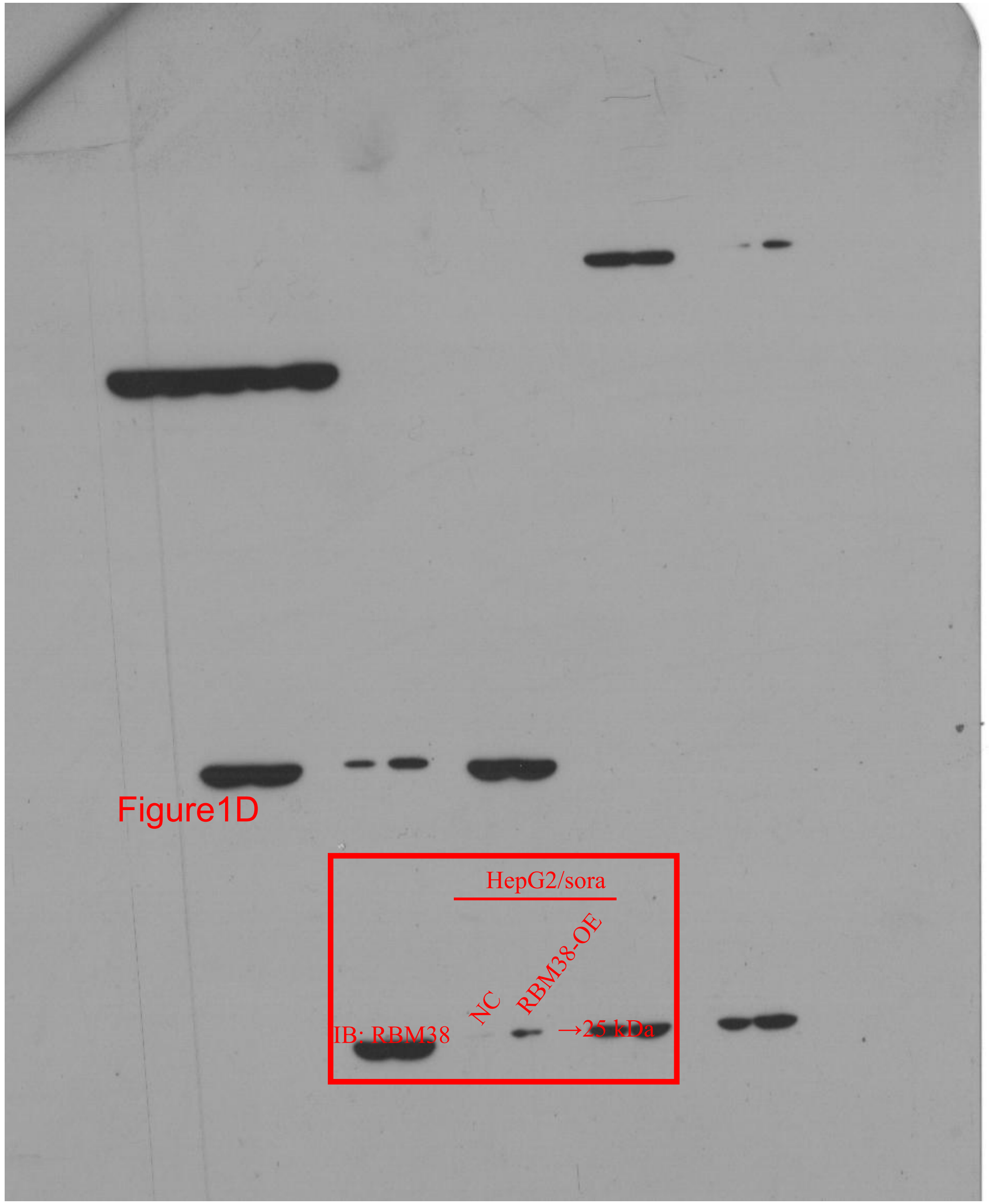

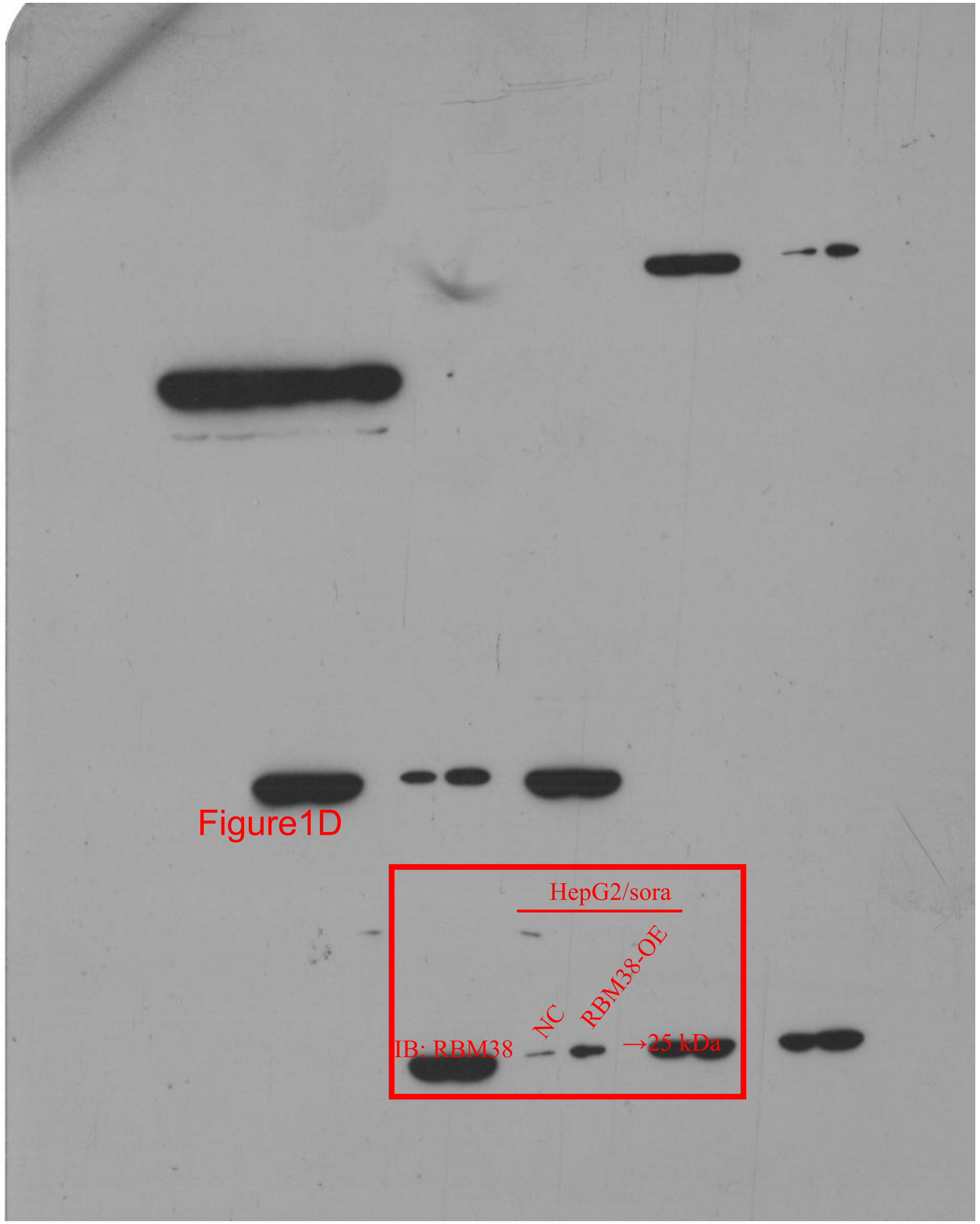

Figure 1D. Overexpression (OE) efficiency of RBM38 in PCLs and drug-resistant HCC cells based on western blot analysis, compared with the negative control (NC).

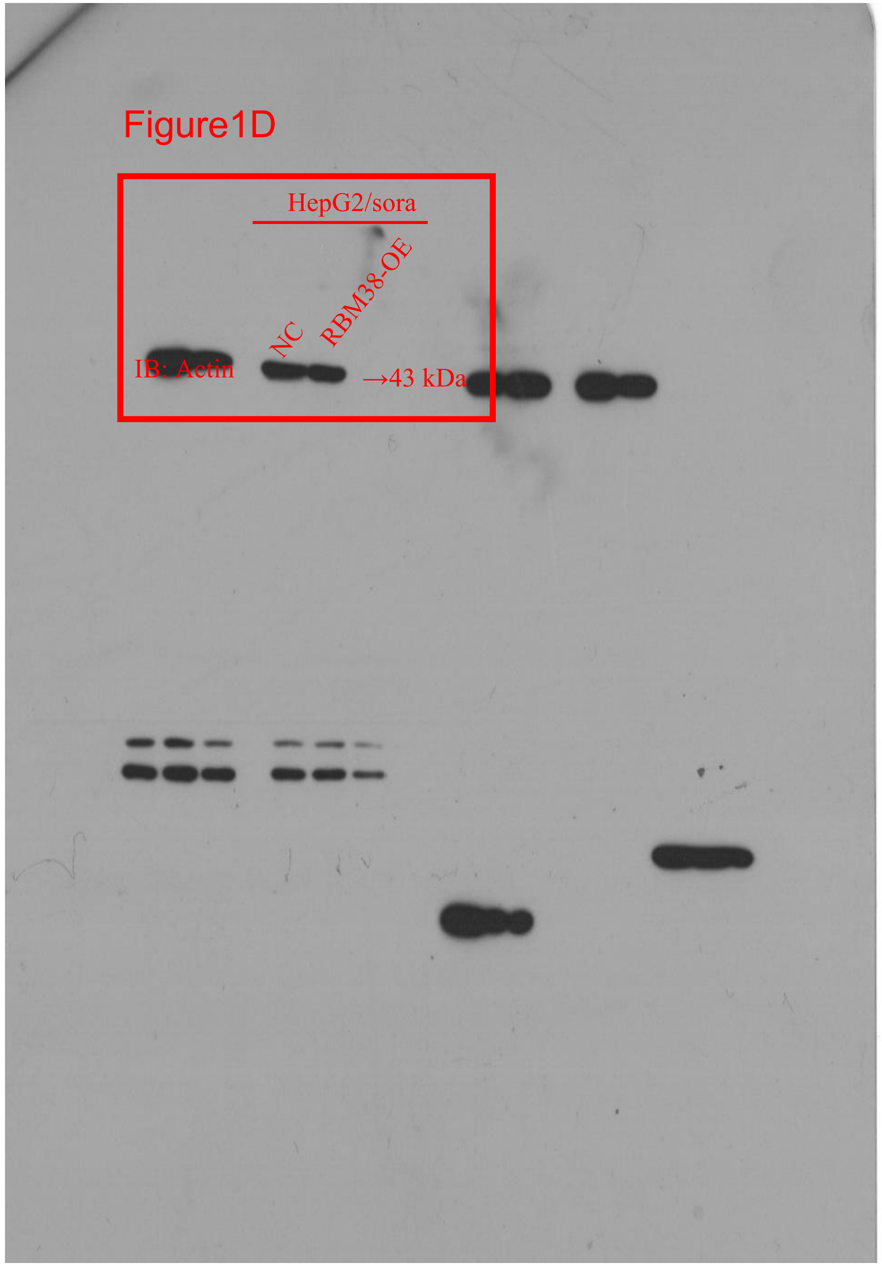

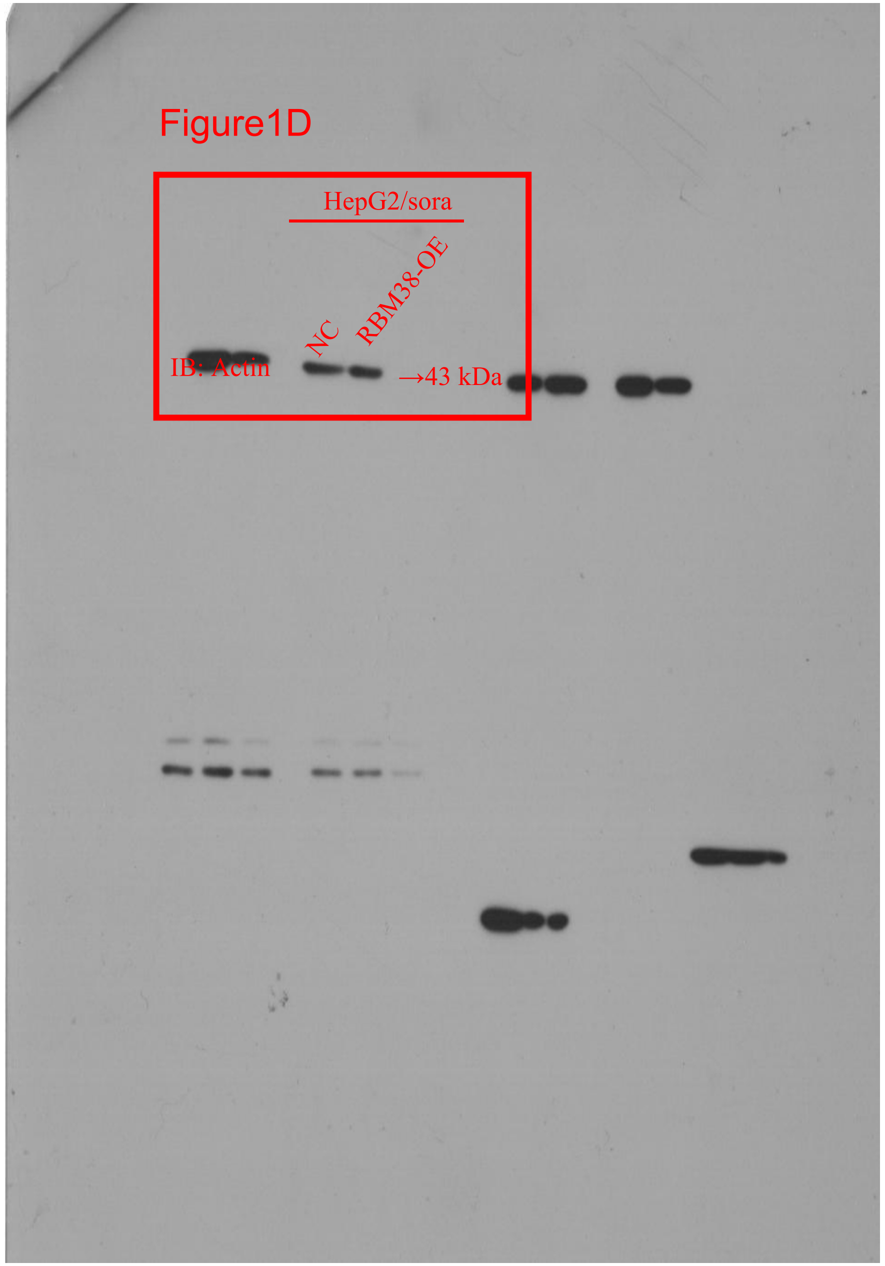

Figure 1F. Western blot analysis of P-GP, MRP1, and ABCG2 levels in 7721/sora-NC, 7721/sora-RBM38-OE, HepG2/sora-NC, and HepG2/sora-RBM38-OE cells.

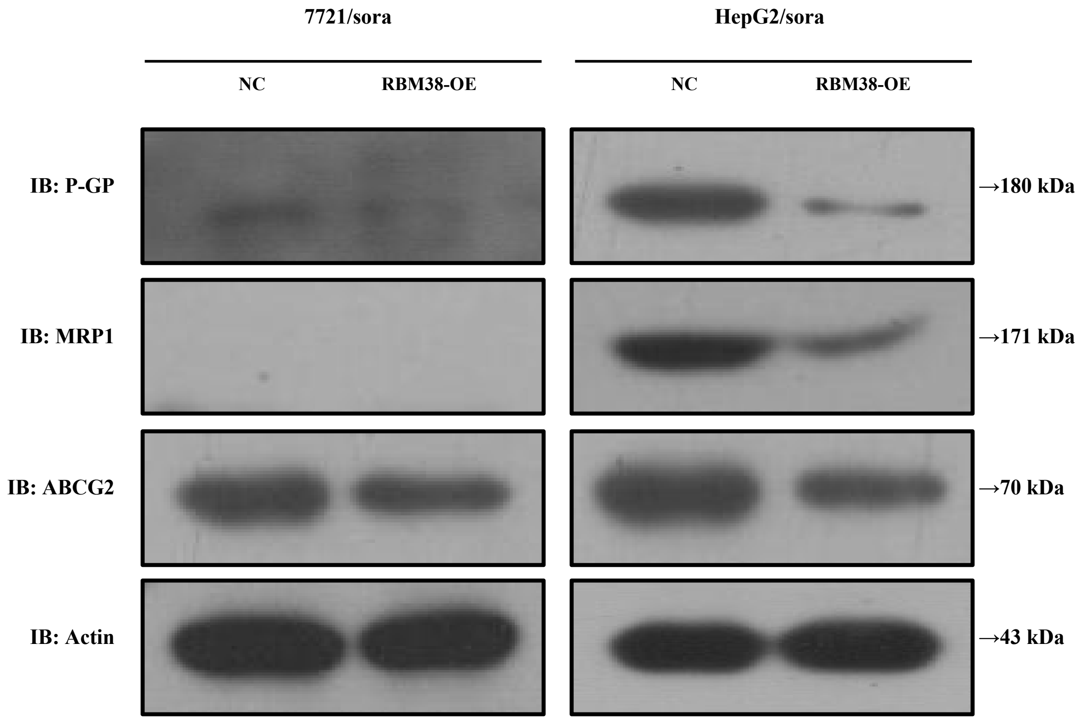

Figure 1F. Western blot analysis of P-GP, MRP1, and ABCG2 levels in 7721/sora-NC, 7721/sora-RBM38-OE, HepG2/sora-NC, and HepG2/sora-RBM38-OE cells.

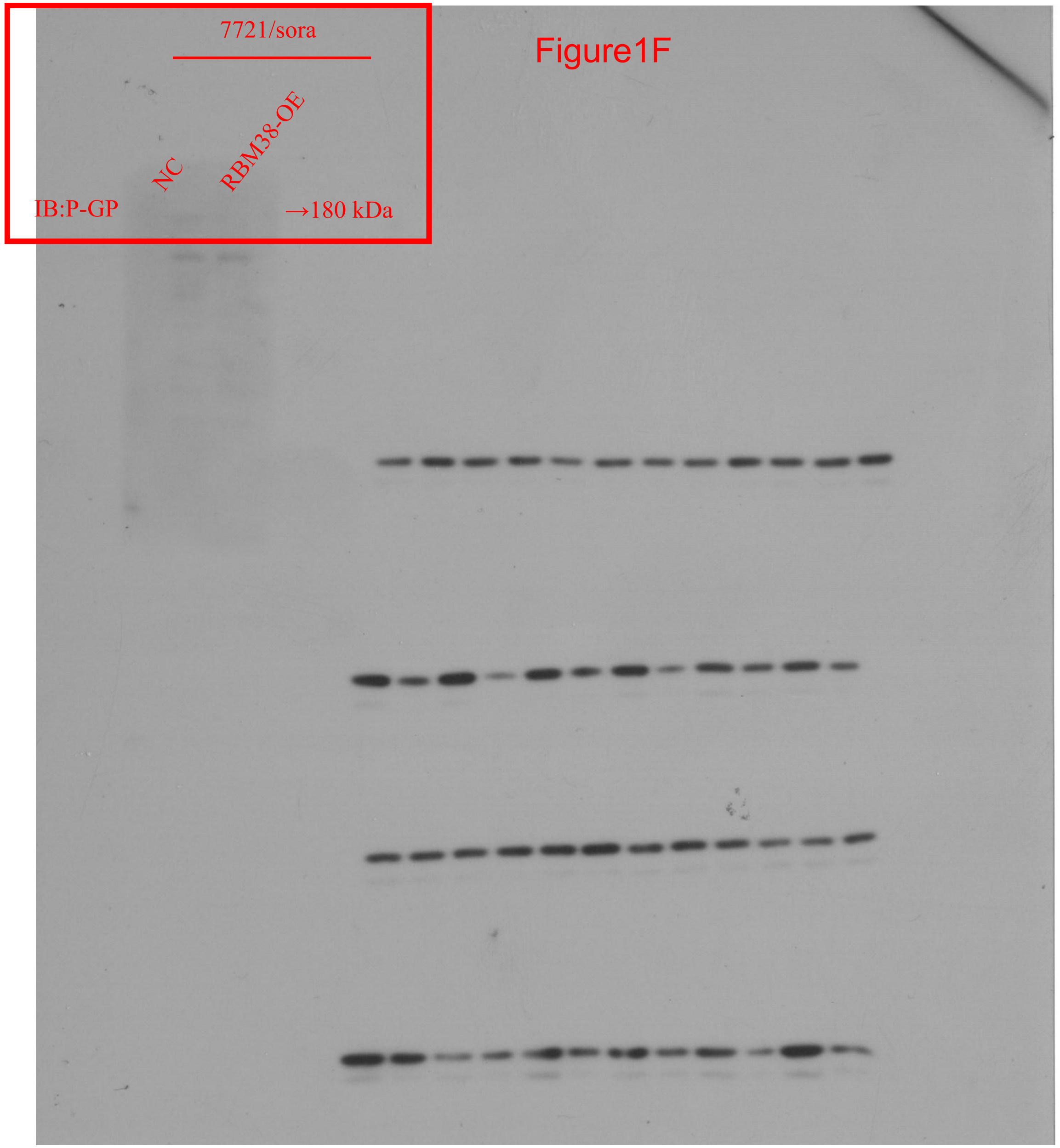

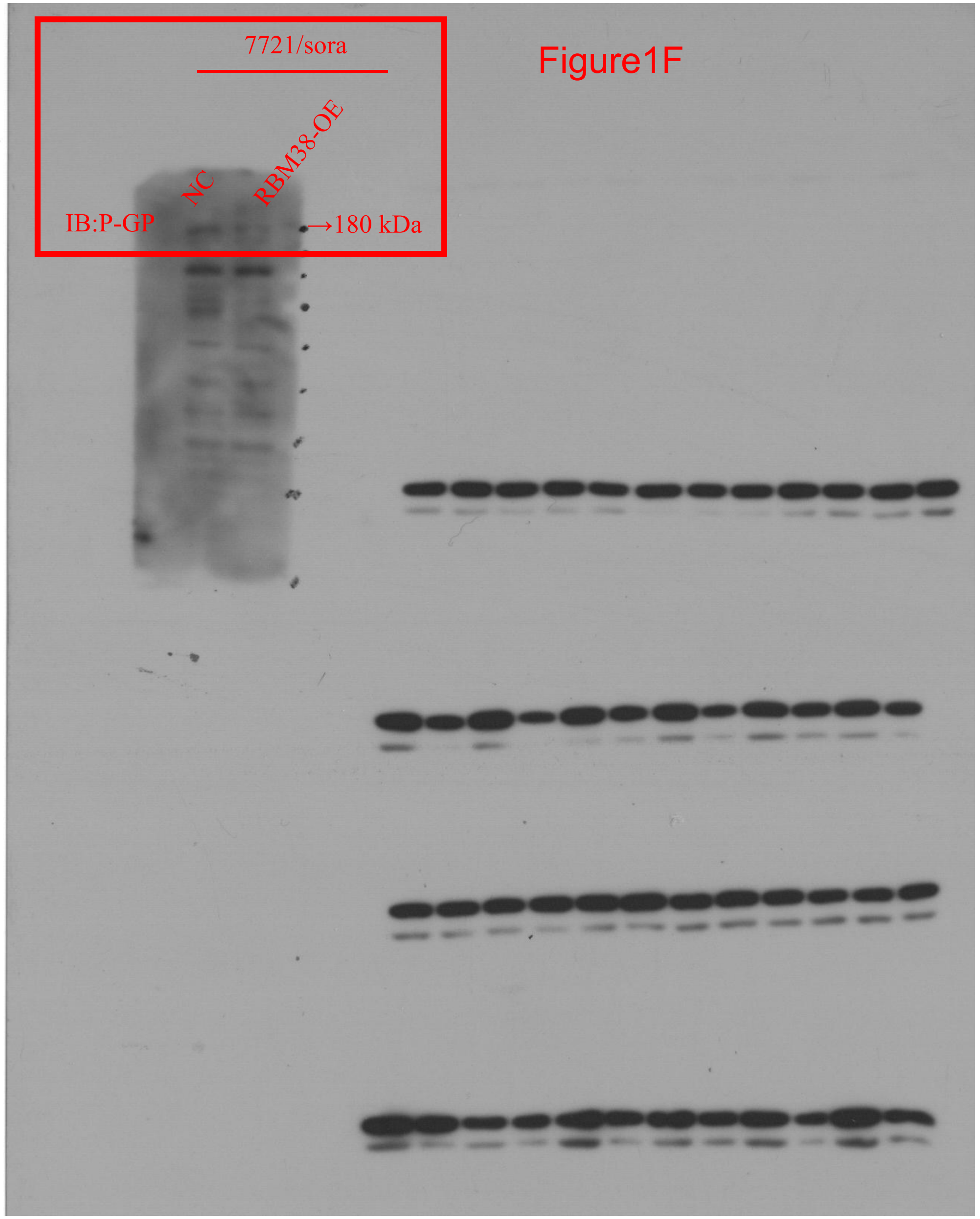

Figure 1F. Western blot analysis of P-GP, MRP1, and ABCG2 levels in 7721/sora-NC, 7721/sora-RBM38-OE, HepG2/sora-NC, and HepG2/sora-RBM38-OE cells.

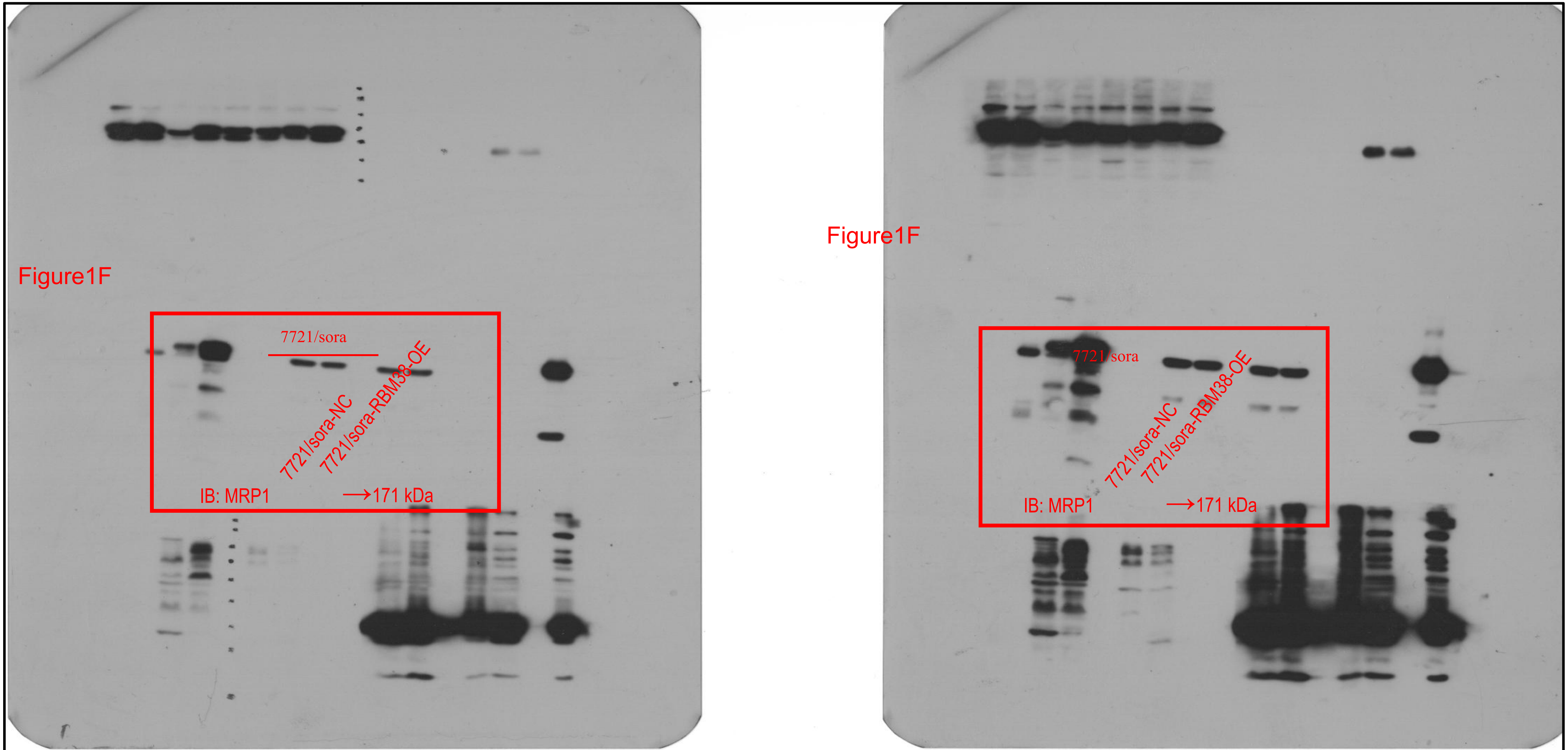

Figure 1F. Western blot analysis of P-GP, MRP1, and ABCG2 levels in 7721/sora-NC, 7721/sora-RBM38-OE, HepG2/sora-NC, and HepG2/sora-RBM38-OE cells.

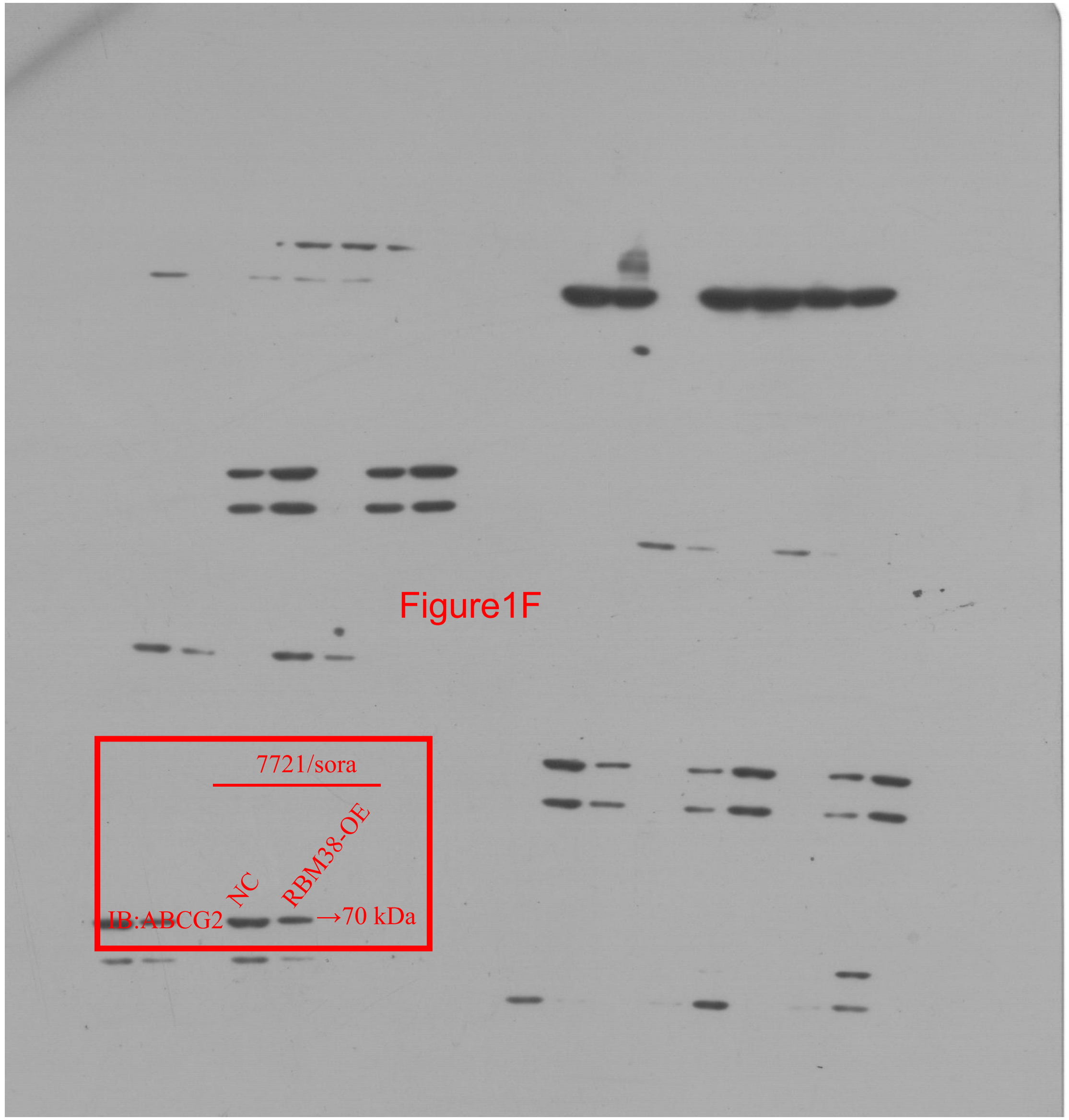

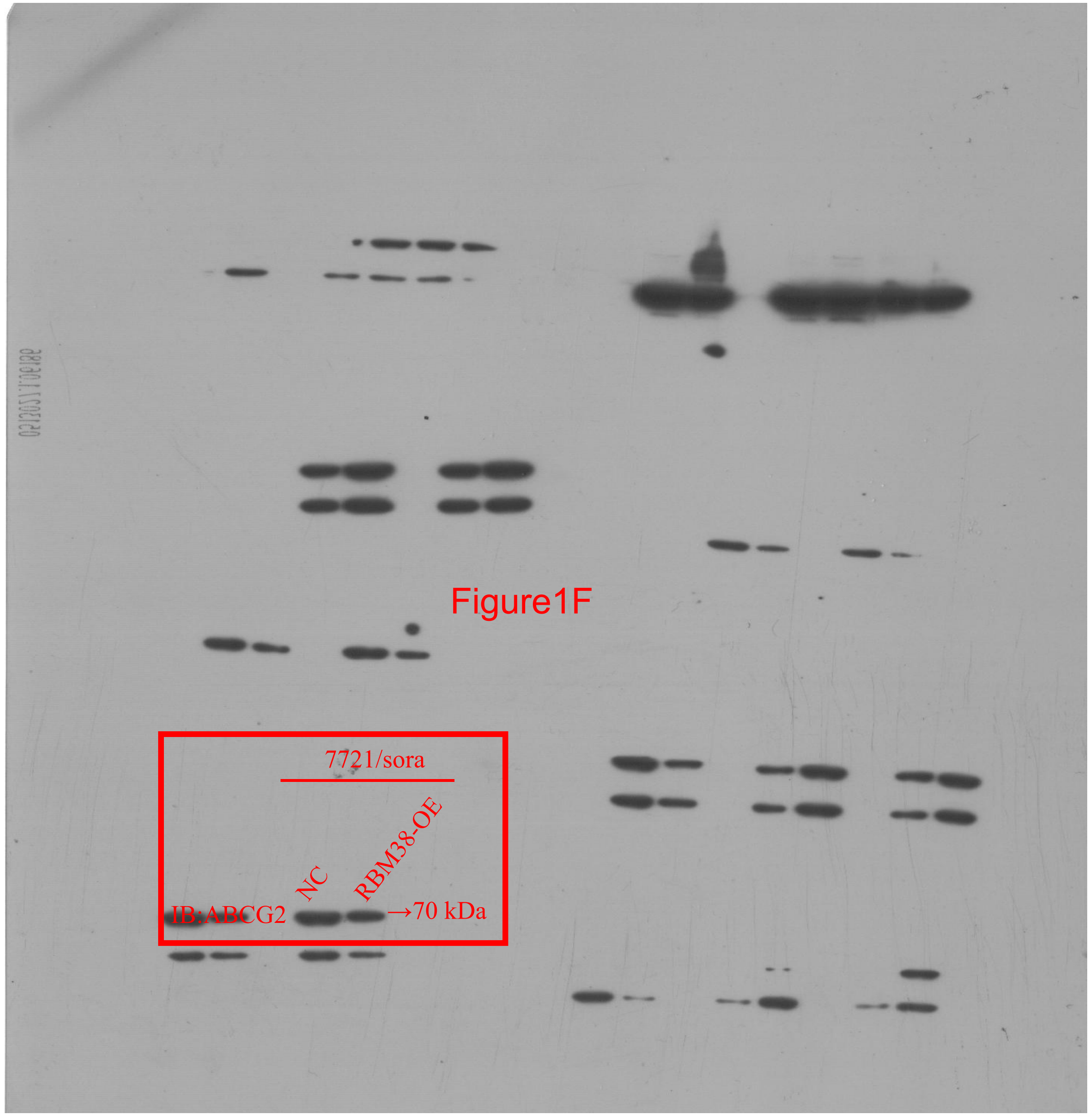

Figure 1F. Western blot analysis of P-GP, MRP1, and ABCG2 levels in 7721/sora-NC, 7721/sora-RBM38-OE, HepG2/sora-NC, and HepG2/sora-RBM38-OE cells.

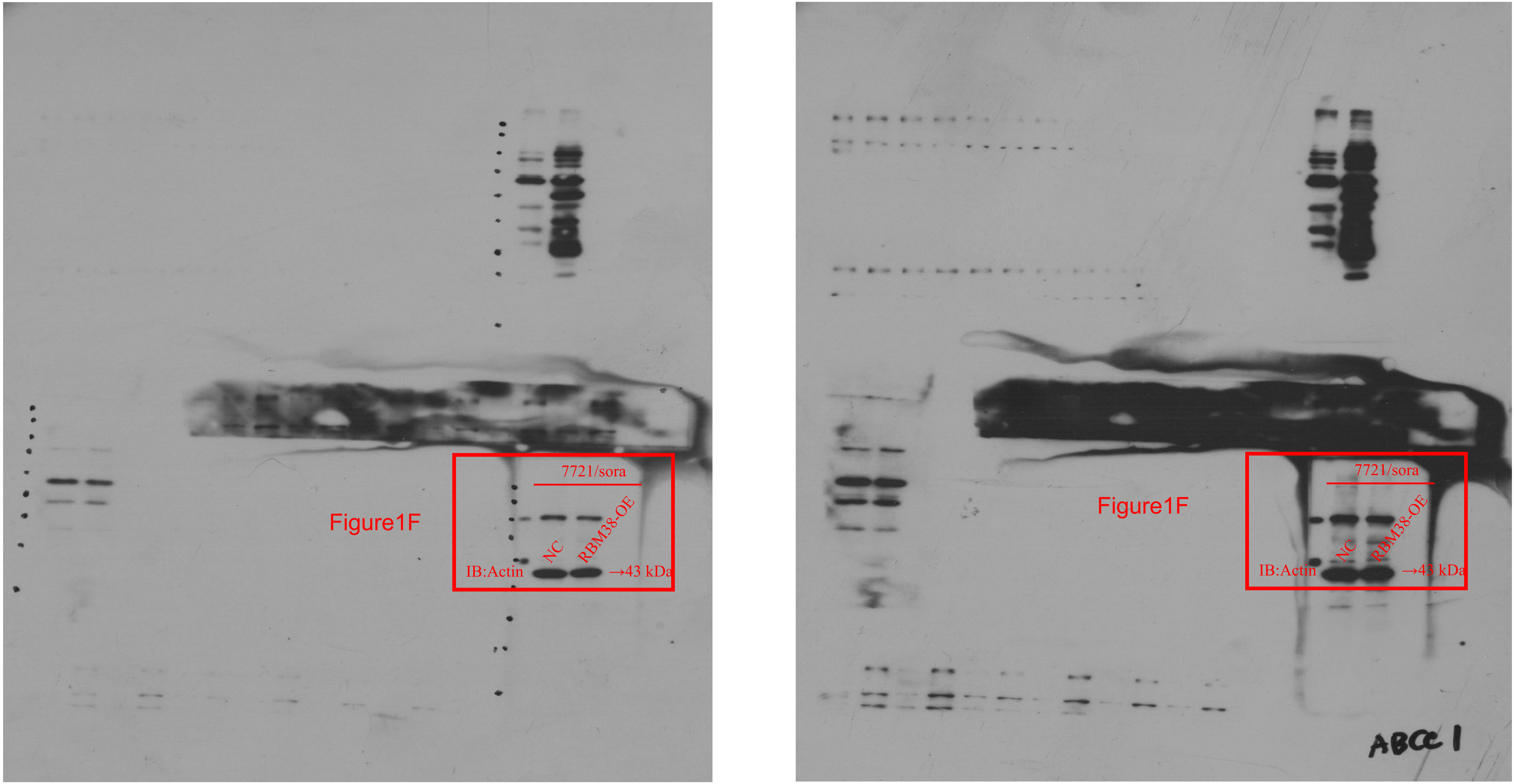

Figure 1F. Western blot analysis of P-GP, MRP1, and ABCG2 levels in 7721/sora-NC, 7721/sora-RBM38-OE, HepG2/sora-NC, and HepG2/sora-RBM38-OE cells.

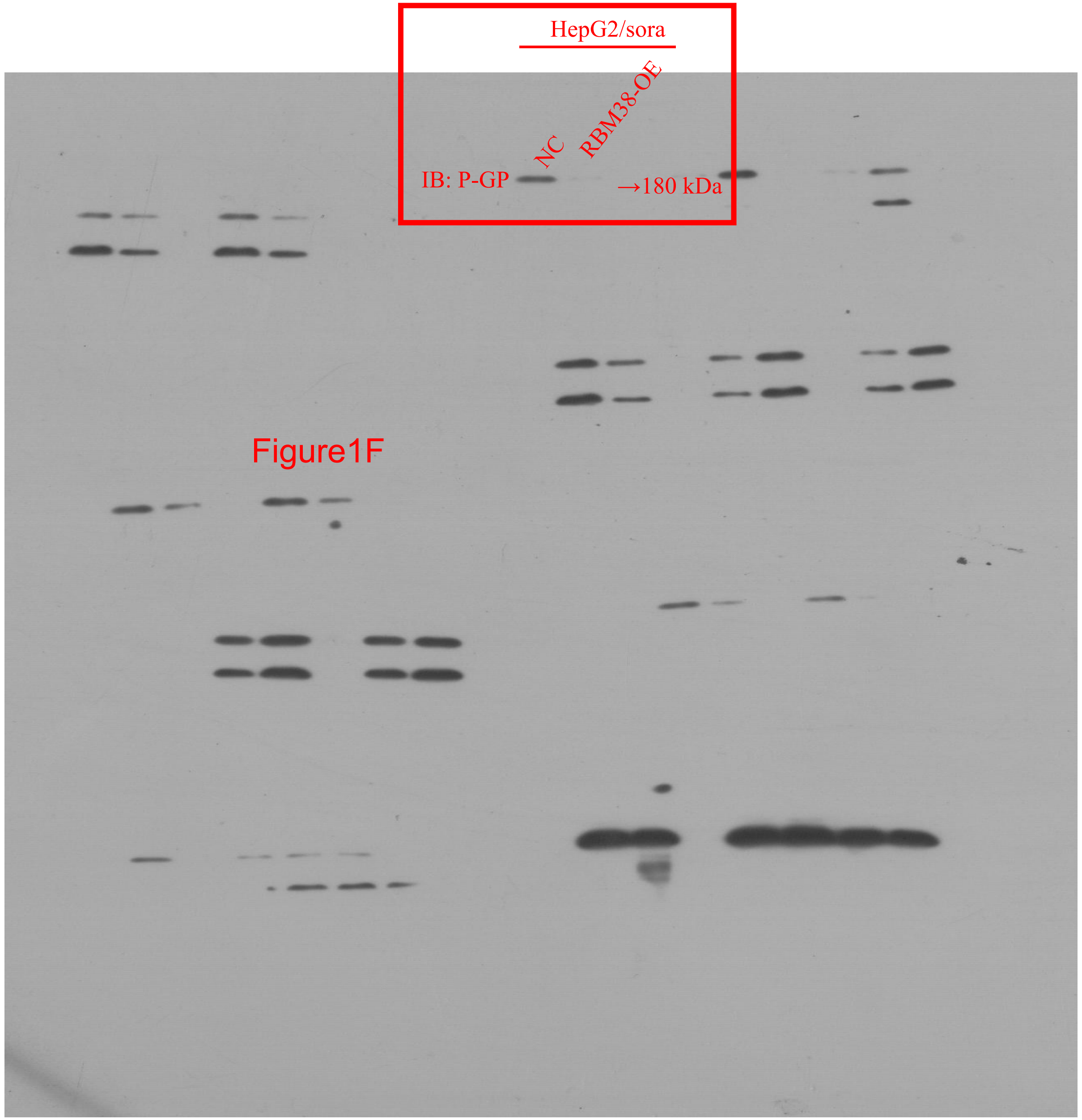

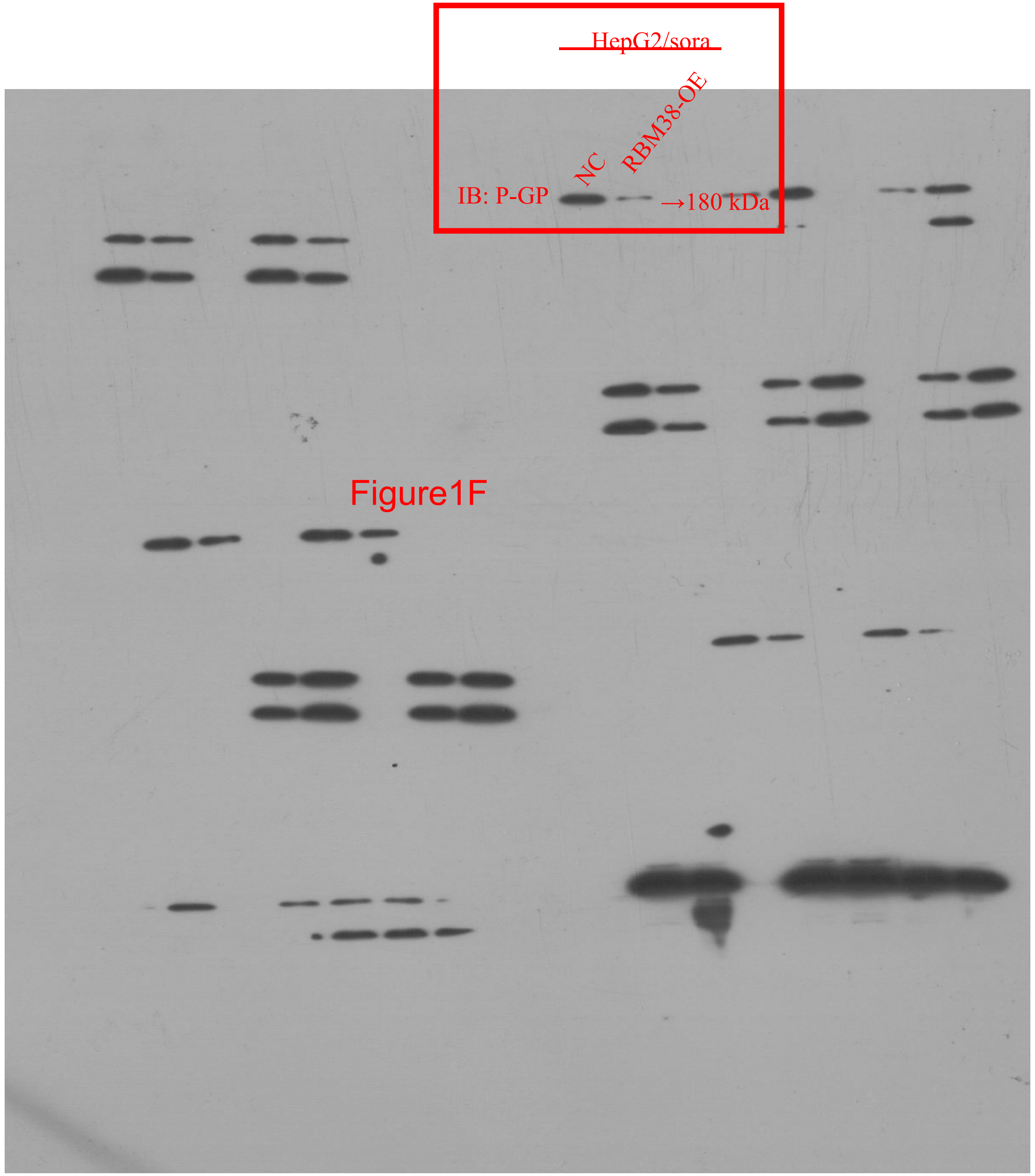

Figure 1F. Western blot analysis of P-GP, MRP1, and ABCG2 levels in 7721/sora-NC, 7721/sora-RBM38-OE, HepG2/sora-NC, and HepG2/sora-RBM38-OE cells.

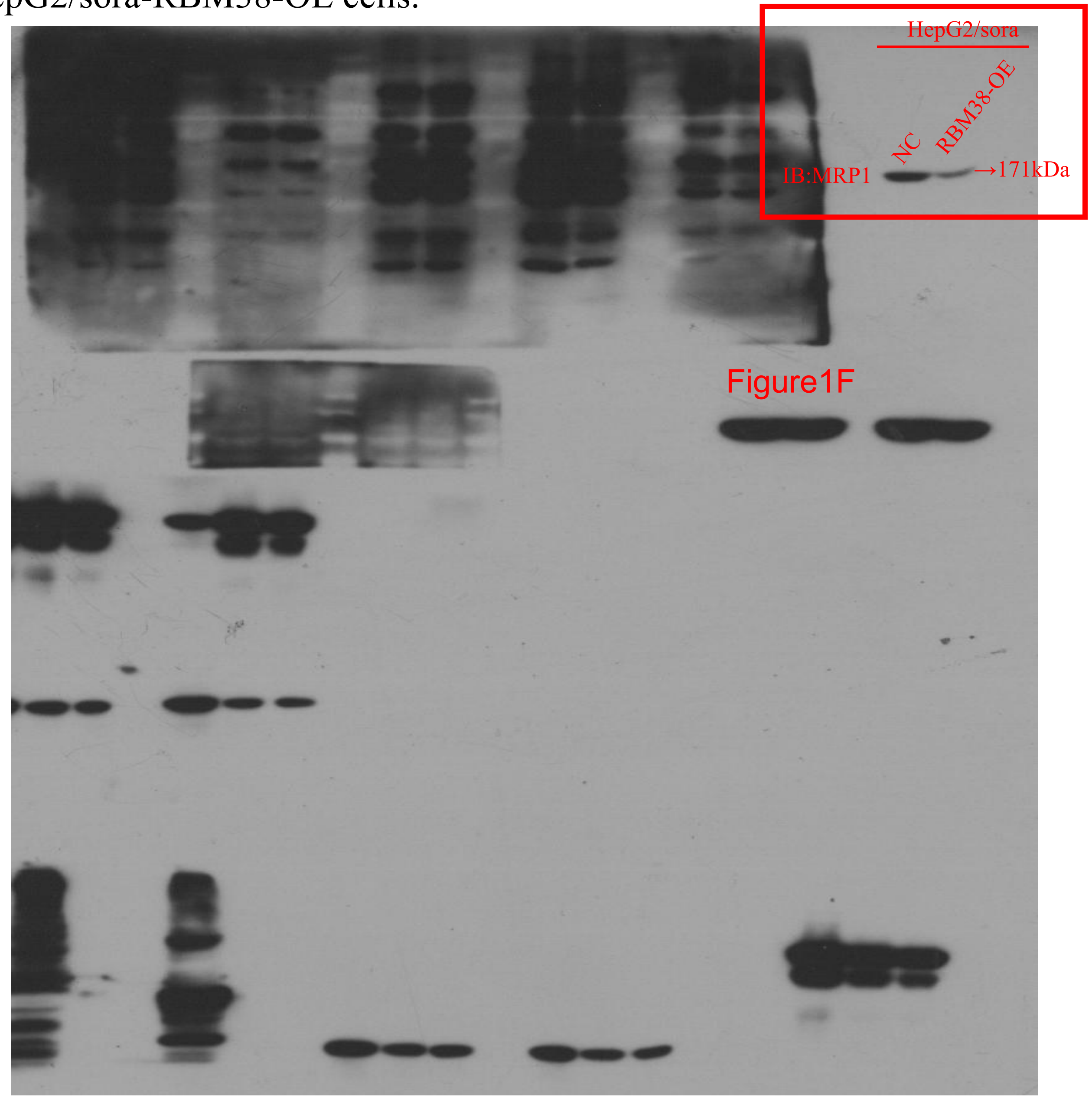

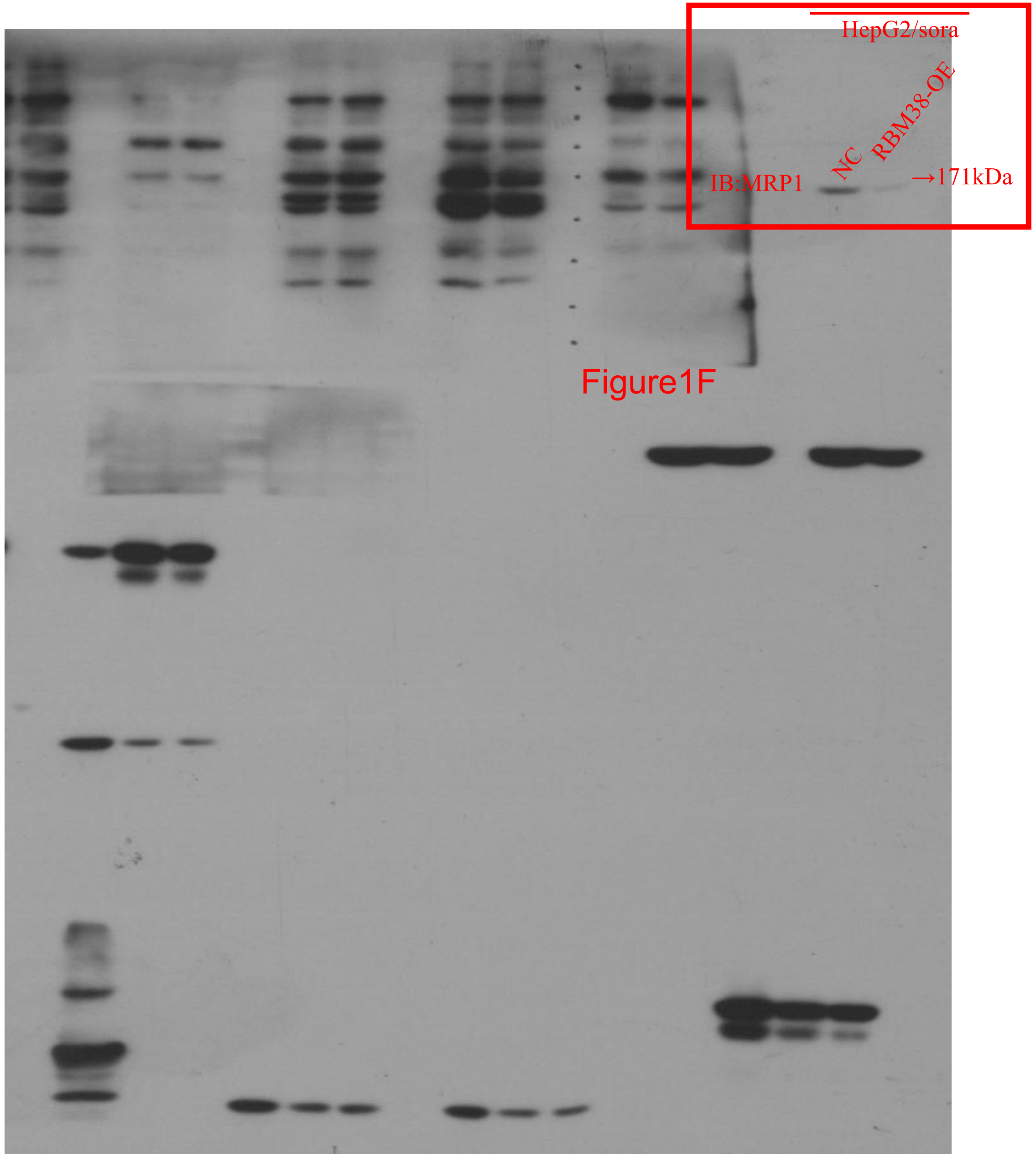

Figure 1F. Western blot analysis of P-GP, MRP1, and ABCG2 levels in 7721/sora-NC, 7721/sora-RBM38-OE, HepG2/sora-NC, and HepG2/sora-RBM38-OE cells.

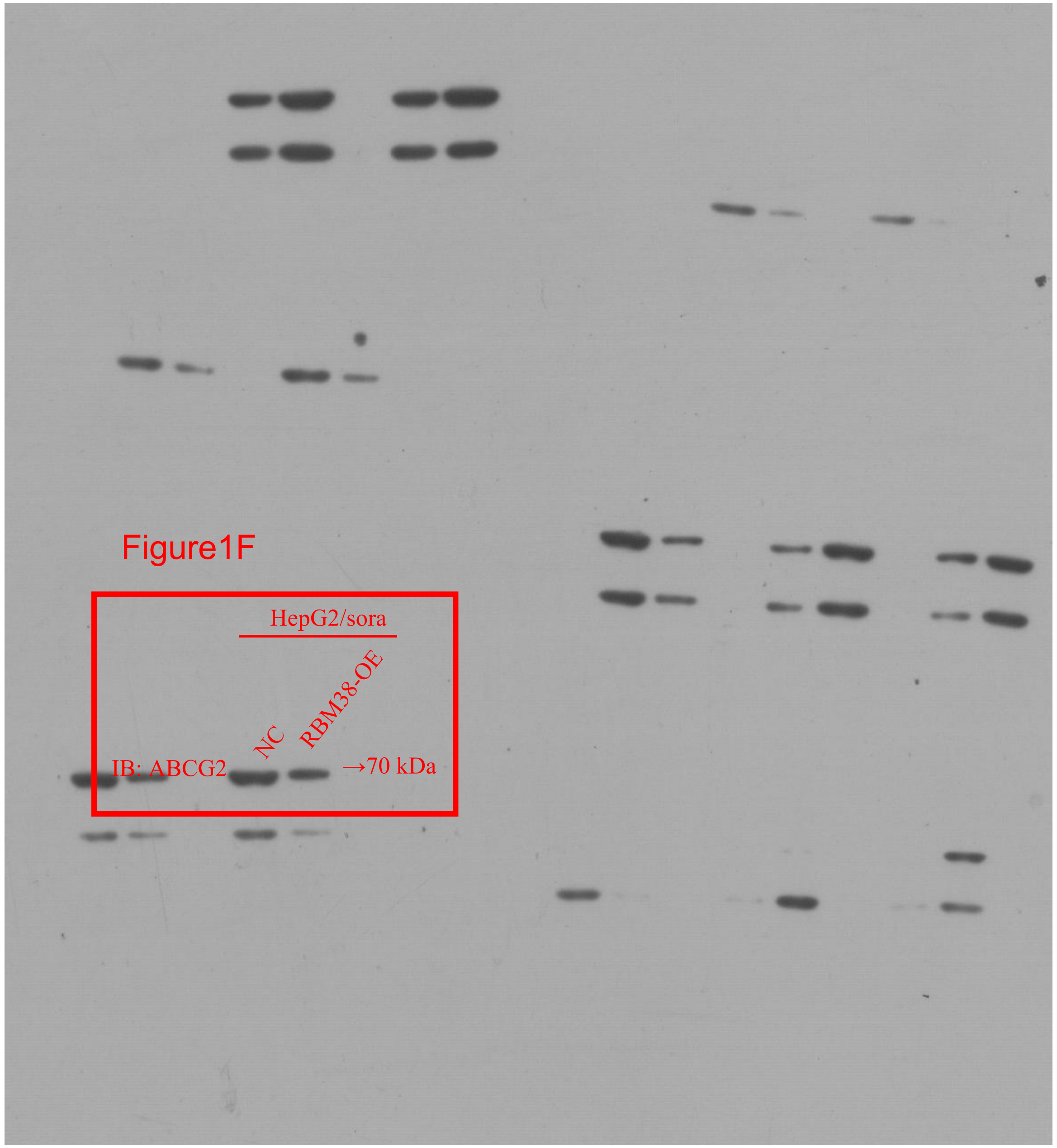

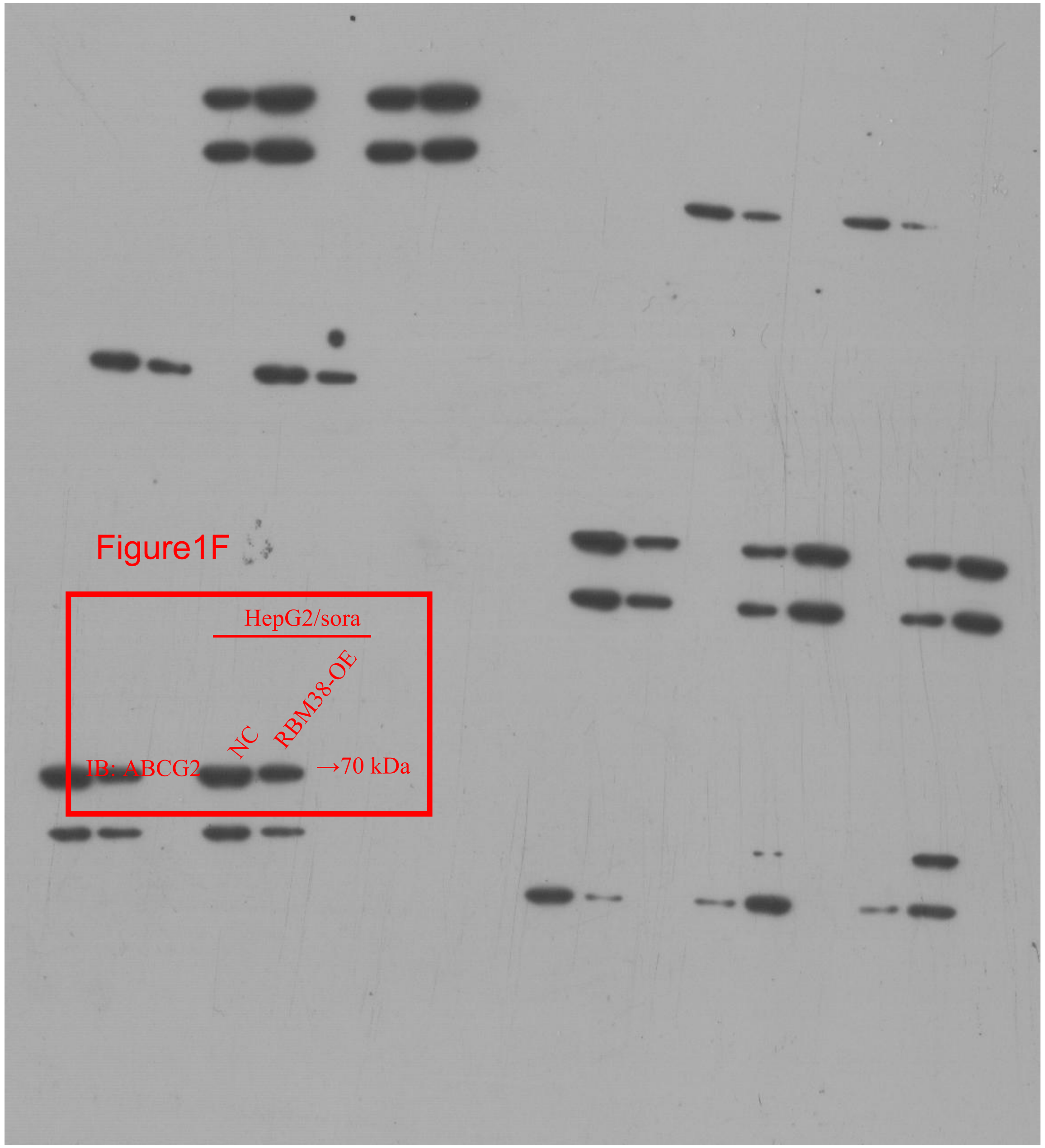

Figure 1F. Western blot analysis of P-GP, MRP1, and ABCG2 levels in 7721/sora-NC, 7721/sora-RBM38-OE, HepG2/sora-NC, and HepG2/sora-RBM38-OE cells.

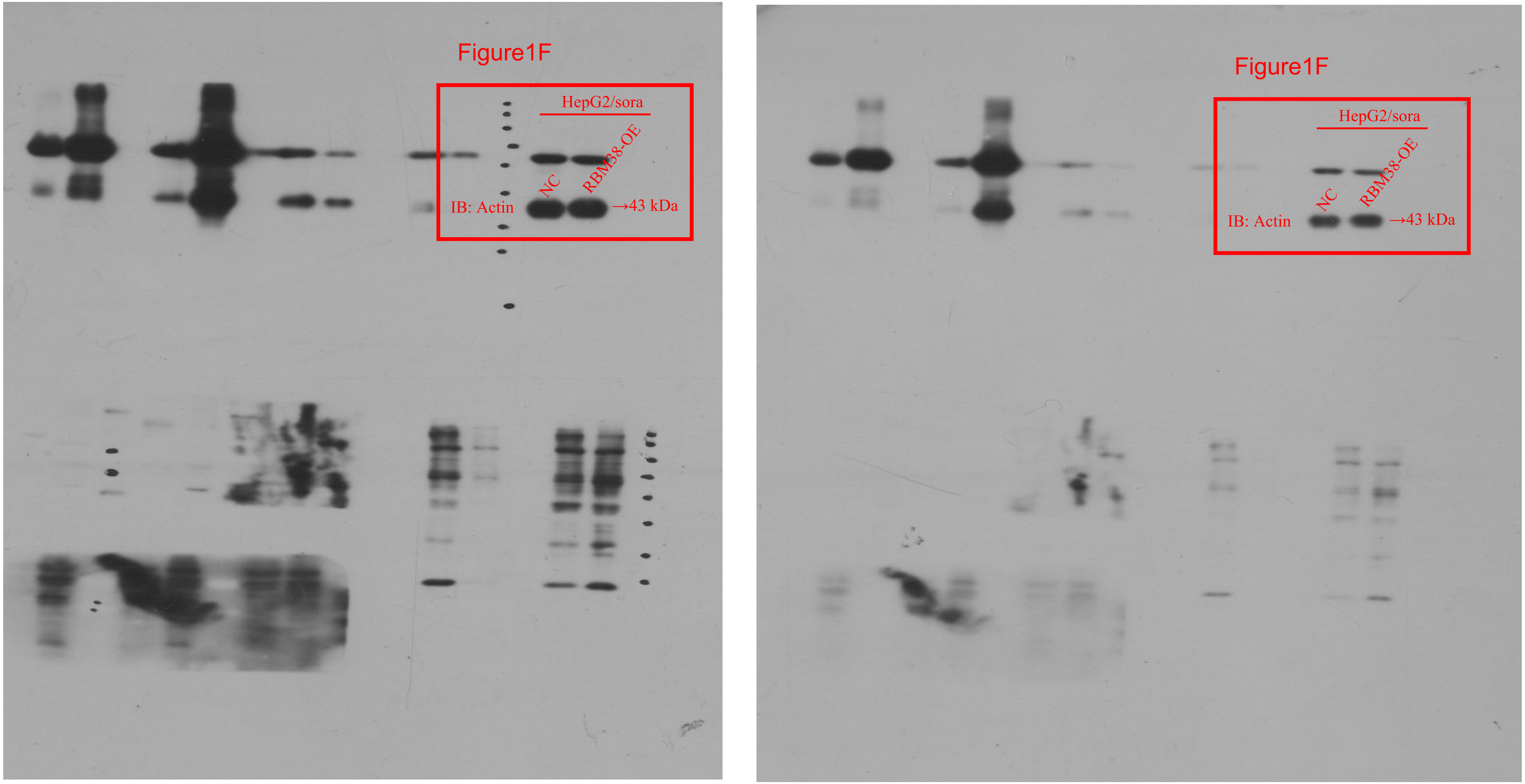

Figure 3

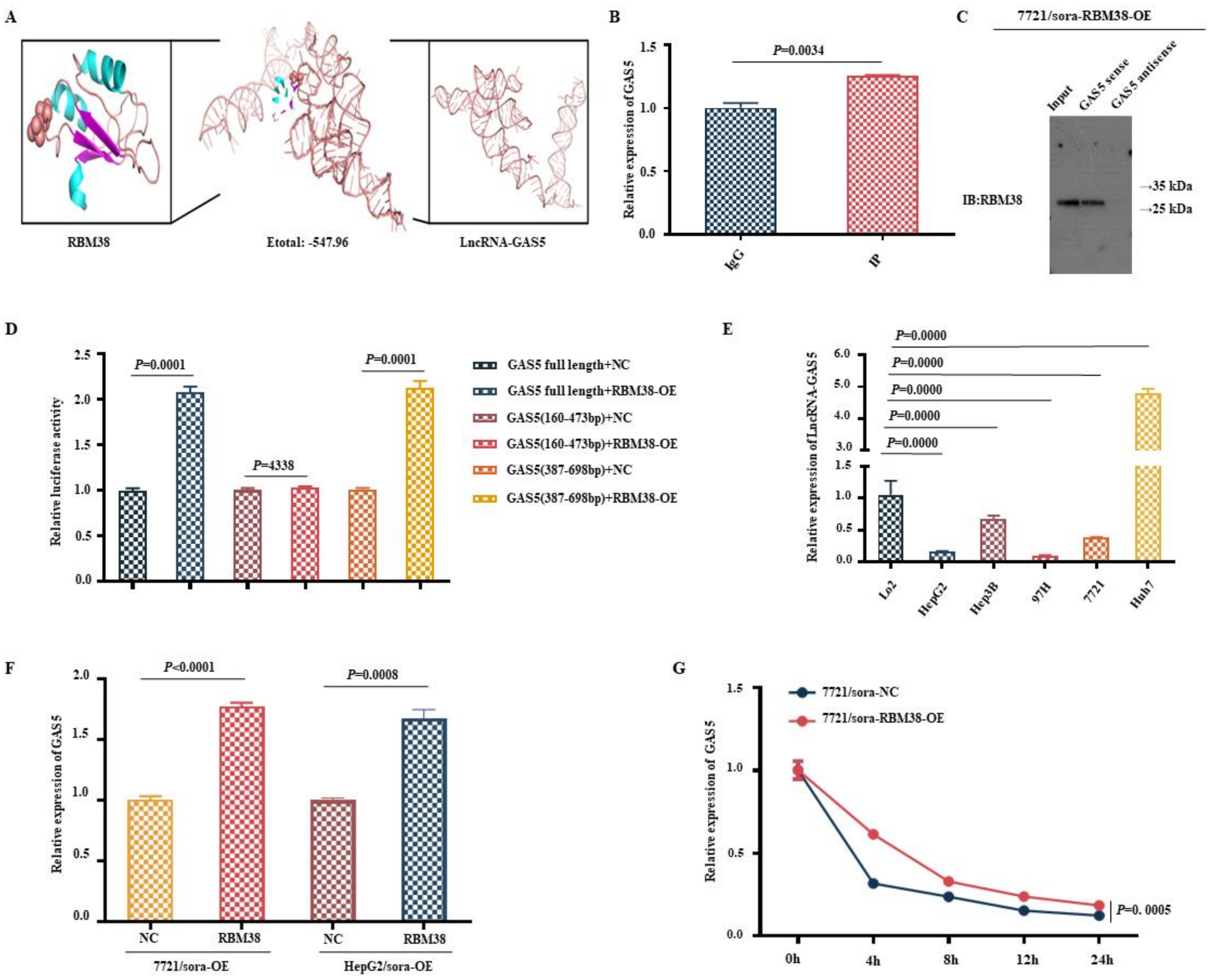
A
RBM38
Etotal: -547.96
LncRNA-GAS5
B
P=0.0034
Relative expression of GAS5
1.5
1.0
0.5
0.0
IgG
IP
C
7721/sora-RBM38-OE
Input
GAS5 sense
GAS5 antisense
IB:RBM38
→35 kDa
→25 kDa
D
Relative luciferase activity
2.5
2.0
1.5
1.0
0.5
0.0
P=0.0001
P=4338
P=0.0001
GAS5 full length+NC
GAS5 full length+RBM38-OE
GAS5(160-473bp)+NC
GAS5(160-473bp)+RBM38-OE
GAS5(387-698bp)+NC
GAS5(387-698bp)+RBM38-OE
E
Relative expression of LncRNA-GAS5
6.0
5.0
4.0
3.0
1.5
1.0
0.5
0.0
P=0.0000
P=0.0000
P=0.0000
P=0.0000
P=0.0000
Lo2
HepG2
Hep3B
97H
7721
Huh7
F
Relative expression of GAS5
2.0
1.5
1.0
0.5
0.0
P<0.0001
P=0.0008
NC
RBM38
NC
RBM38
7721/sora-OE
HepG2/sora-OE
G
Relative expression of GAS5
1.5
1.0
0.5
0.0
7721/sora-NC
7721/sora-RBM38-OE
P=0. 0005
0h
4h
8h
12h
24h

Figure 3C. RNA pulldown assay in 7721/sora-RBM38-OE cells to validate the interaction between RBM38 and GAS5.

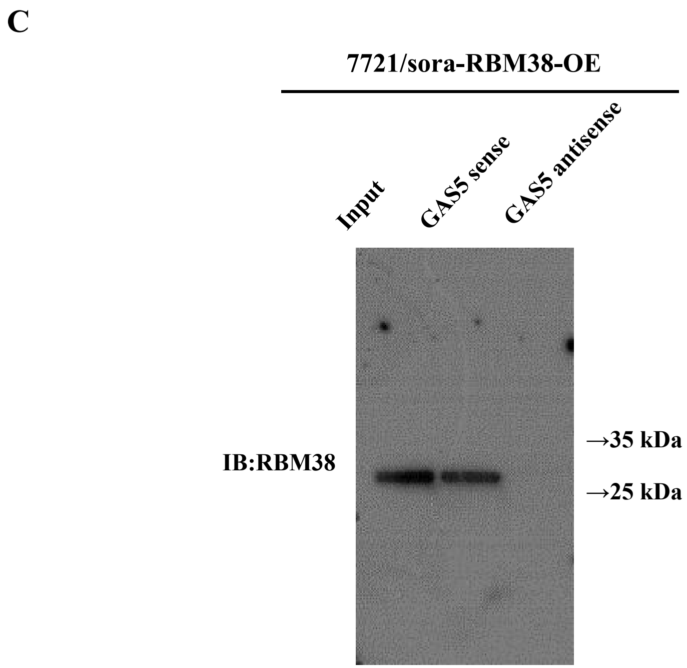

Figure 3C. RNA pulldown assay in 7721/sora-RBM38-OE cells to validate the interaction between RBM38 and GAS5.

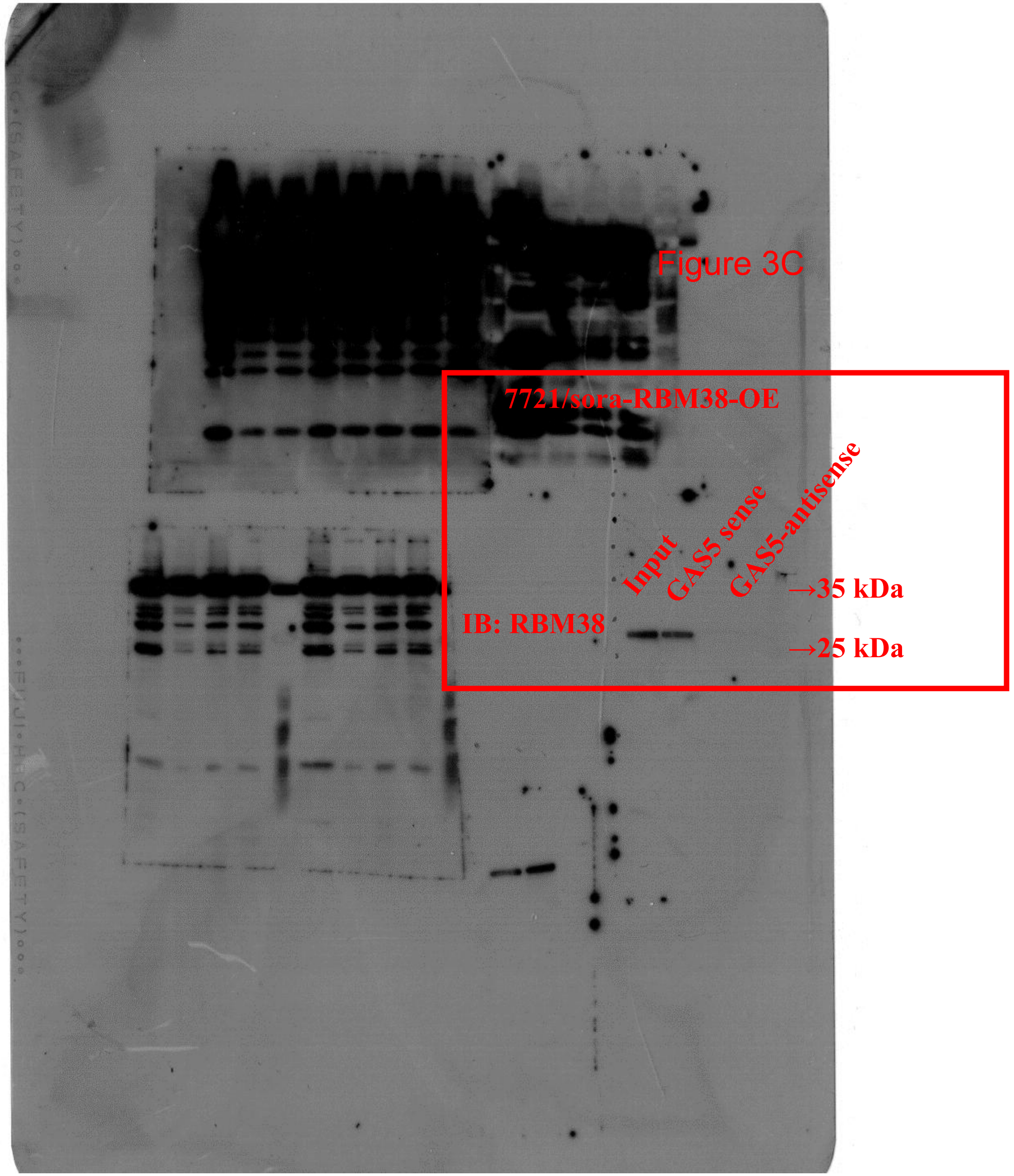

Figure 4

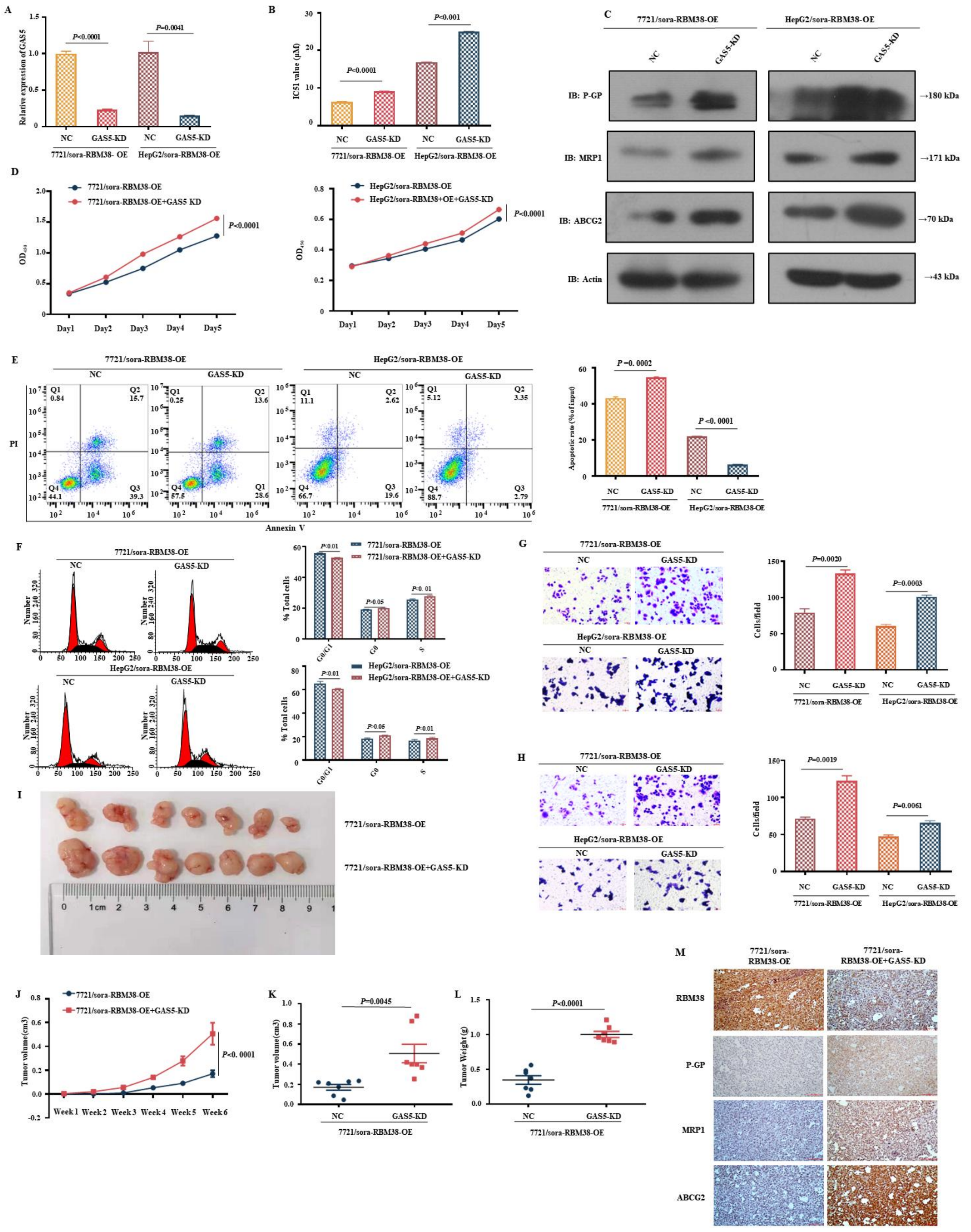

Figure 4C. Western blot analysis of P-GP, MRP1, and ABCG2 levels in 7721/sora-RBM38-OE+NC, 7721/sora-RBM38-OE+GAS5-KD, HepG2/sora-RBM38-OE+NC, and HepG2/sora-RBM38-OE+GAS5-KD cells.

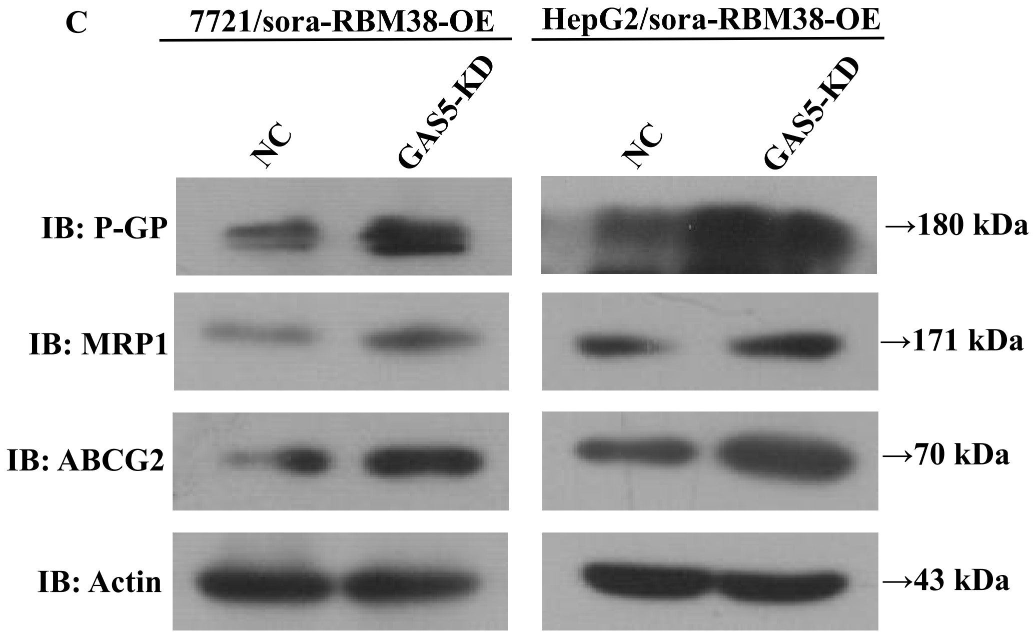

Figure 4C. Western blot analysis of P-GP, MRP1, and ABCG2 levels in 7721/sora-RBM38-OE+NC, 7721/sora-RBM38-OE+GAS5-KD, HepG2/sora-RBM38-OE+NC, and HepG2/sora-RBM38-OE+GAS5-KD cells.

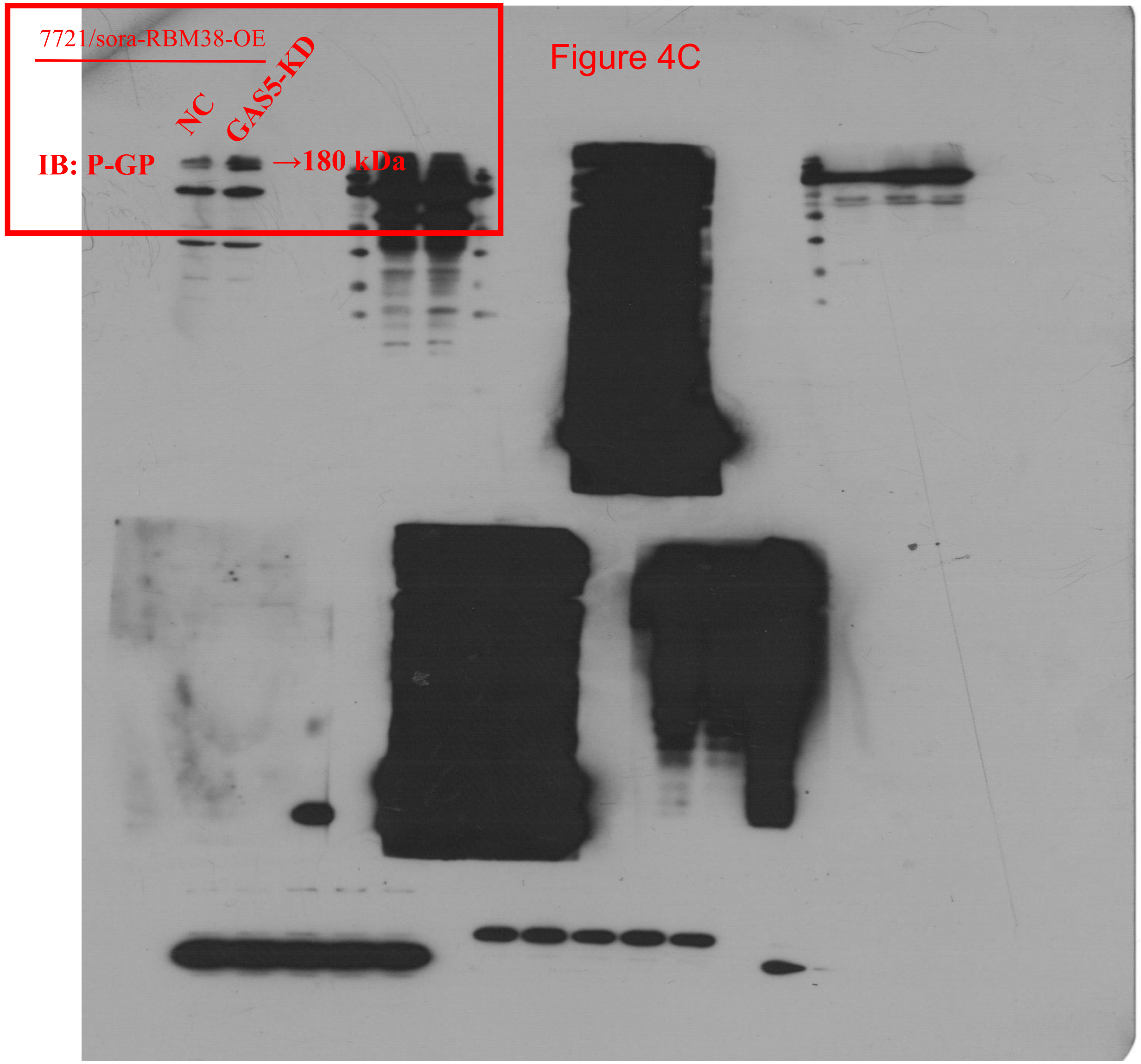

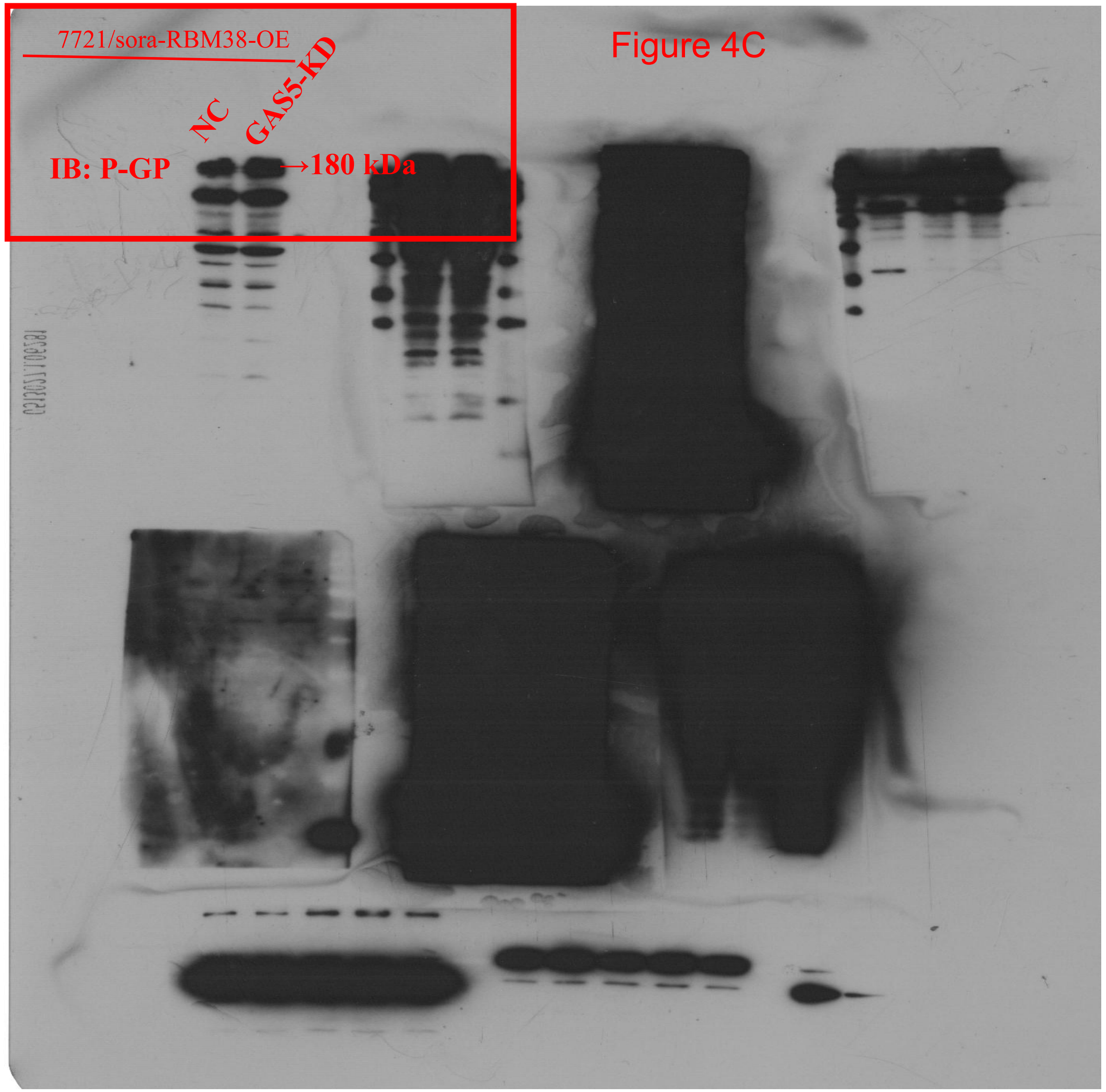

Figure 4C. Western blot analysis of P-GP, MRP1, and ABCG2 levels in 7721/sora-RBM38-OE+NC, 7721/sora-RBM38-OE+GAS5-KD, HepG2/sora-RBM38-OE+NC, and HepG2/sora-RBM38-OE+GAS5-KD cells.

Figure 4C

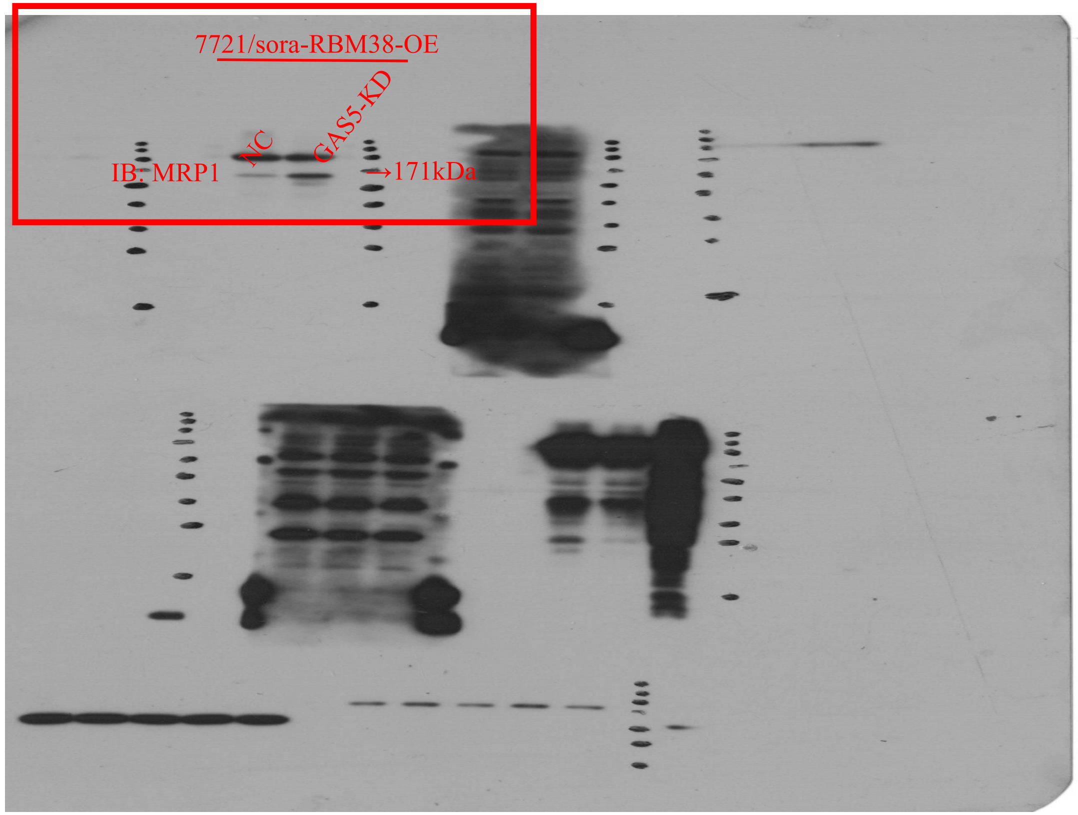

Figure 4C

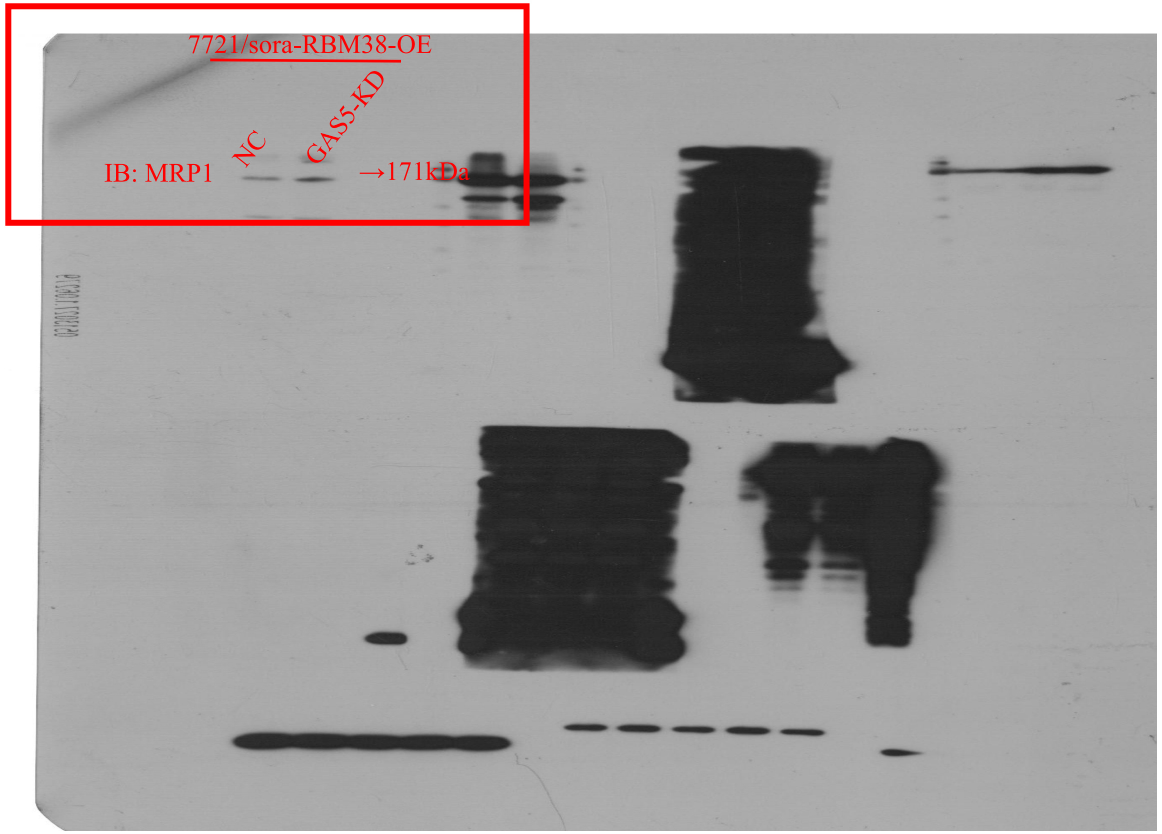

Figure 4C. Western blot analysis of P-GP, MRP1, and ABCG2 levels in 7721/sora-RBM38-OE+NC, 7721/sora-RBM38-OE+GAS5-KD, HepG2/sora-RBM38-OE+NC, and HepG2/sora-RBM38-OE+GAS5-KD cells.

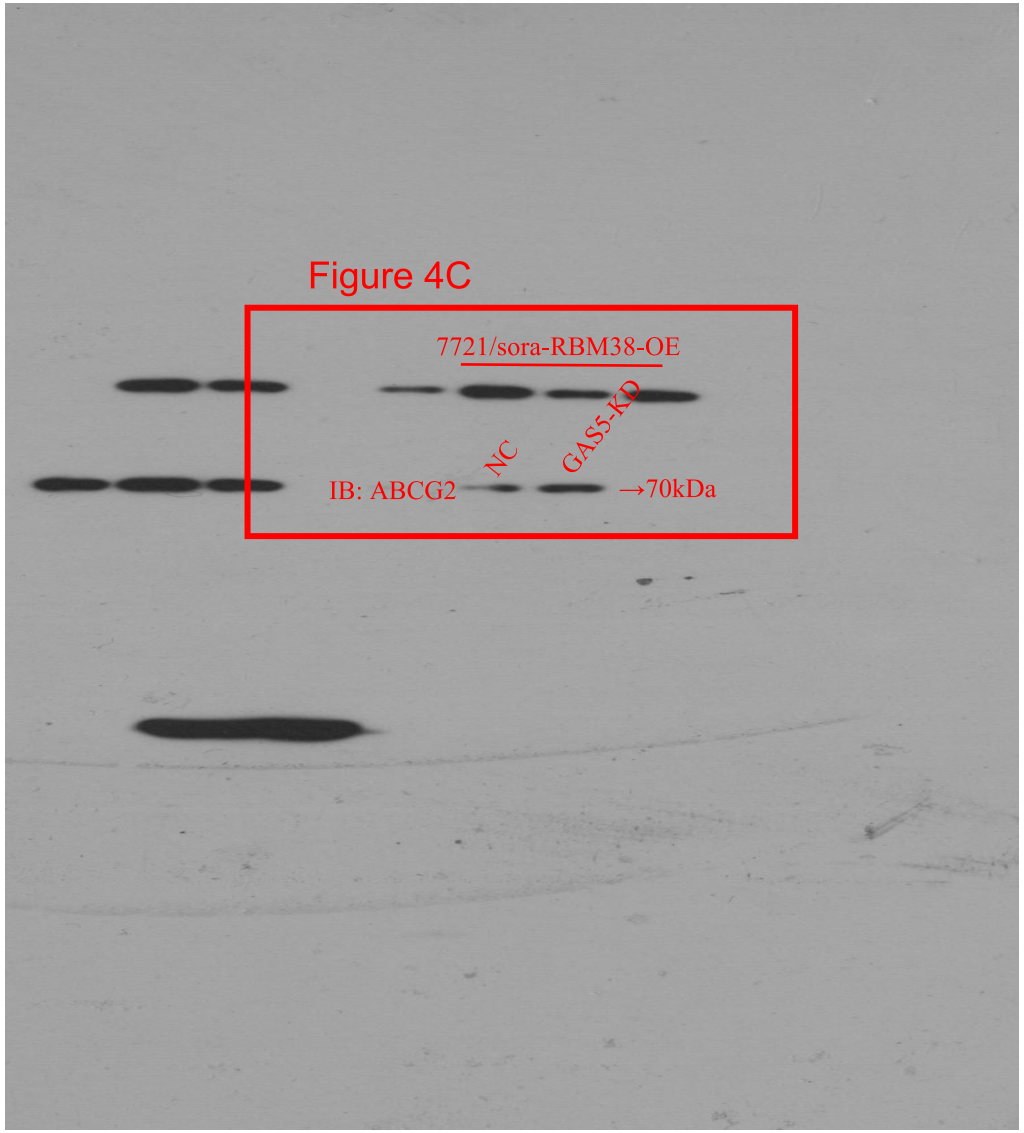

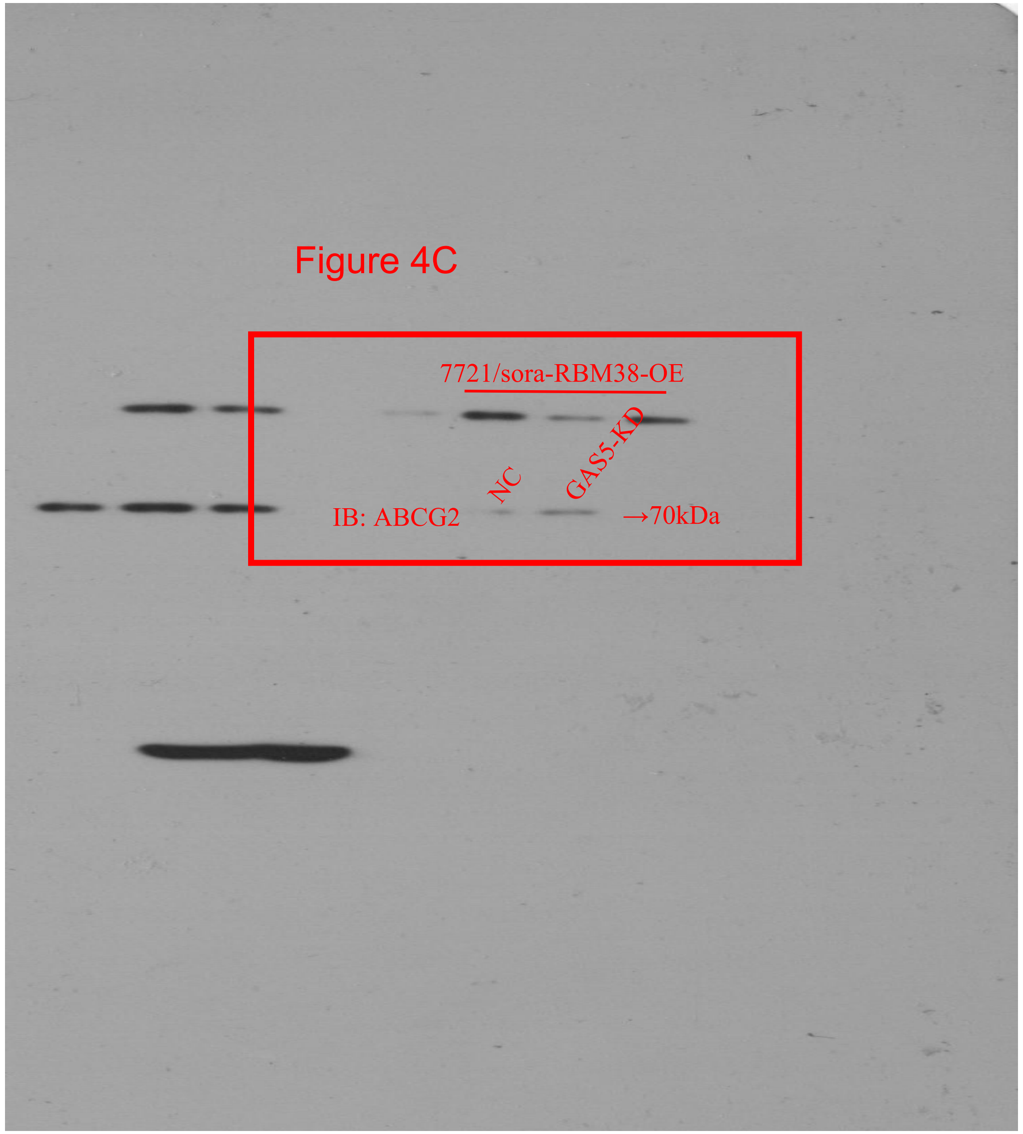

Figure 4C. Western blot analysis of P-GP, MRP1, and ABCG2 levels in 7721/sora-RBM38-OE+NC, 7721/sora-RBM38-OE+GAS5-KD, HepG2/sora-RBM38-OE+NC, and HepG2/sora-RBM38-OE+GAS5-KD cells.

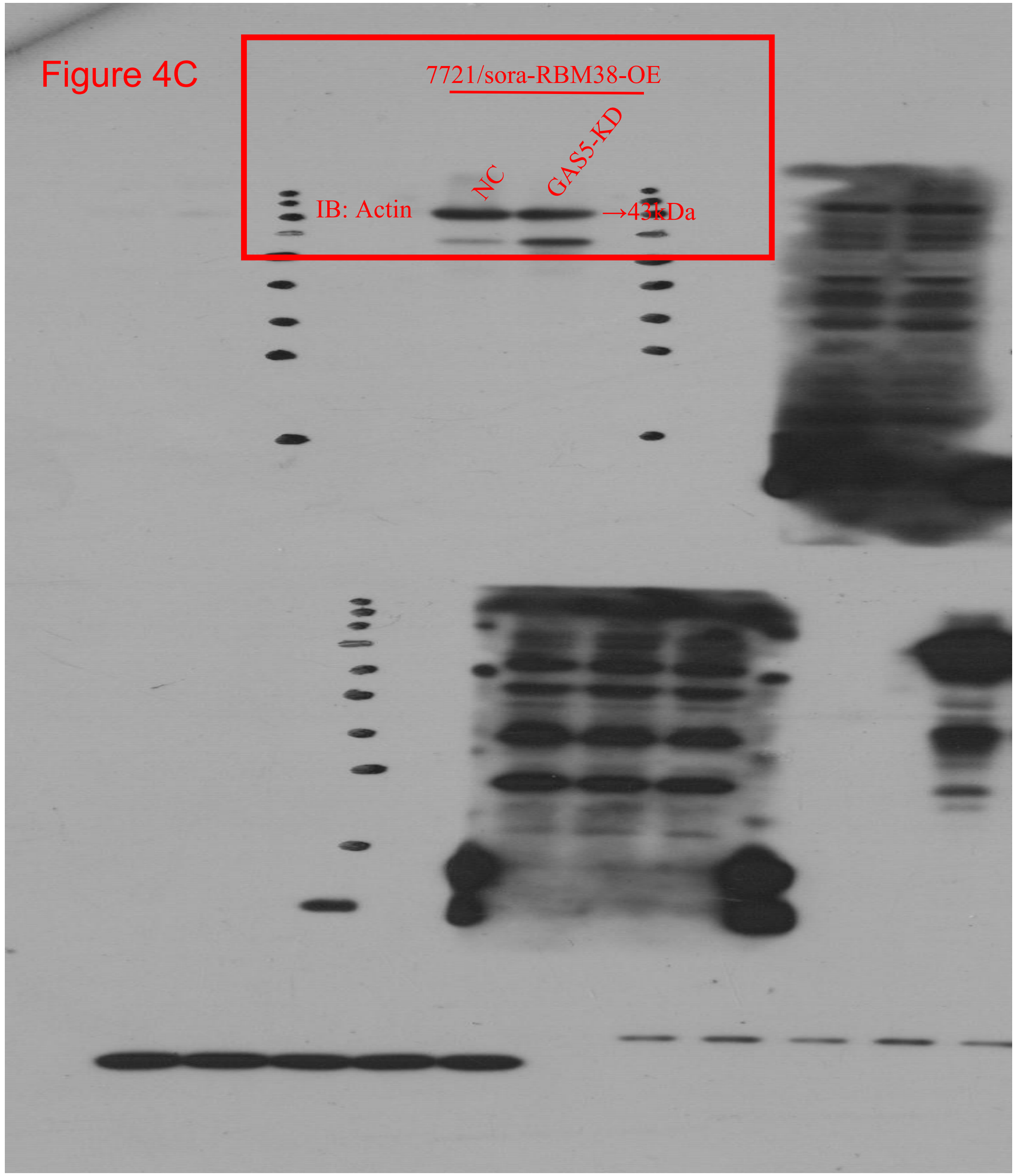

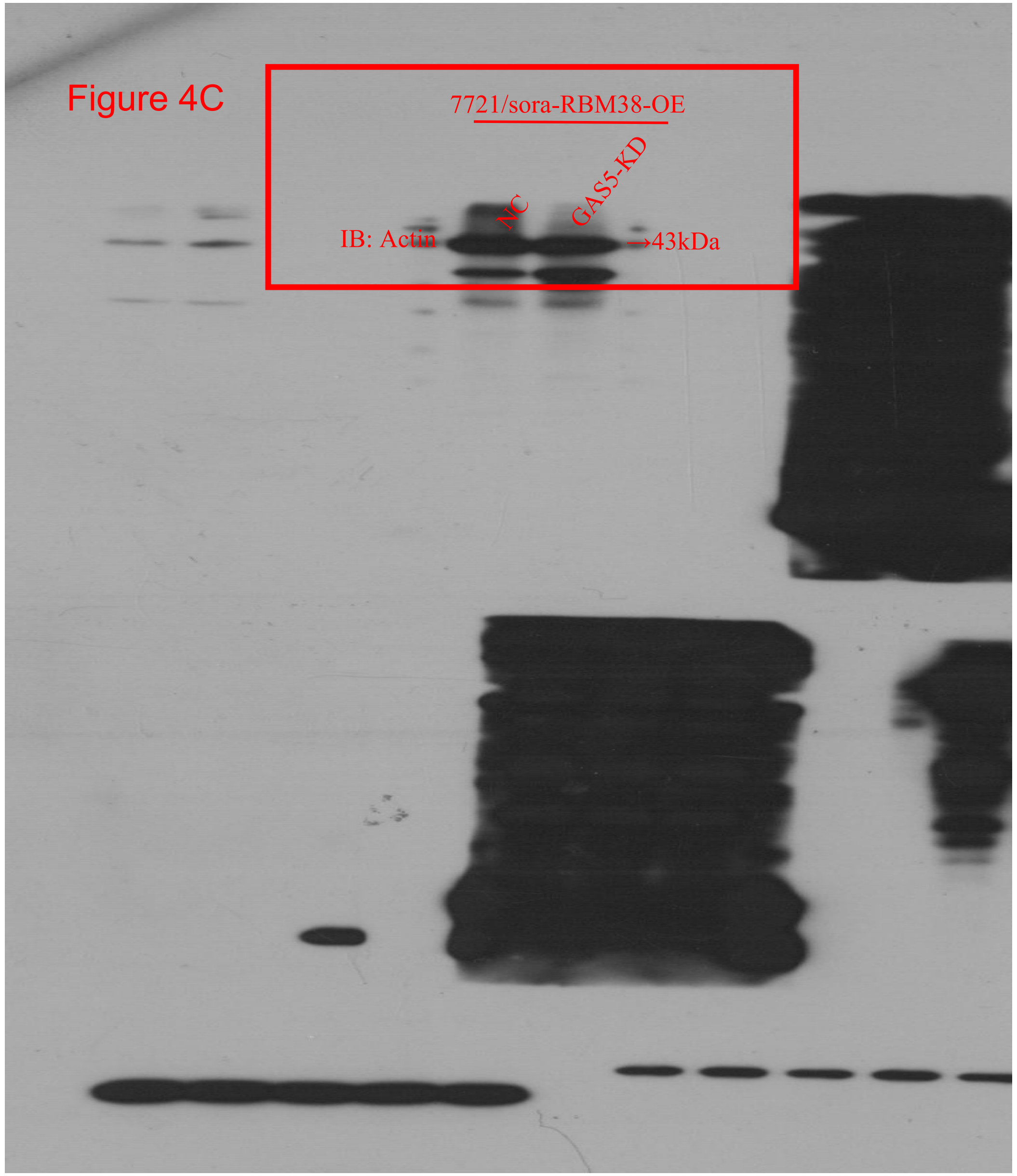

Figure 4C. Western blot analysis of P-GP, MRP1, and ABCG2 levels in 7721/sora-RBM38-OE+NC, 7721/sora-RBM38-OE+GAS5-KD, HepG2/sora-RBM38-OE+NC, and HepG2/sora-RBM38-OE+GAS5-KD cells.

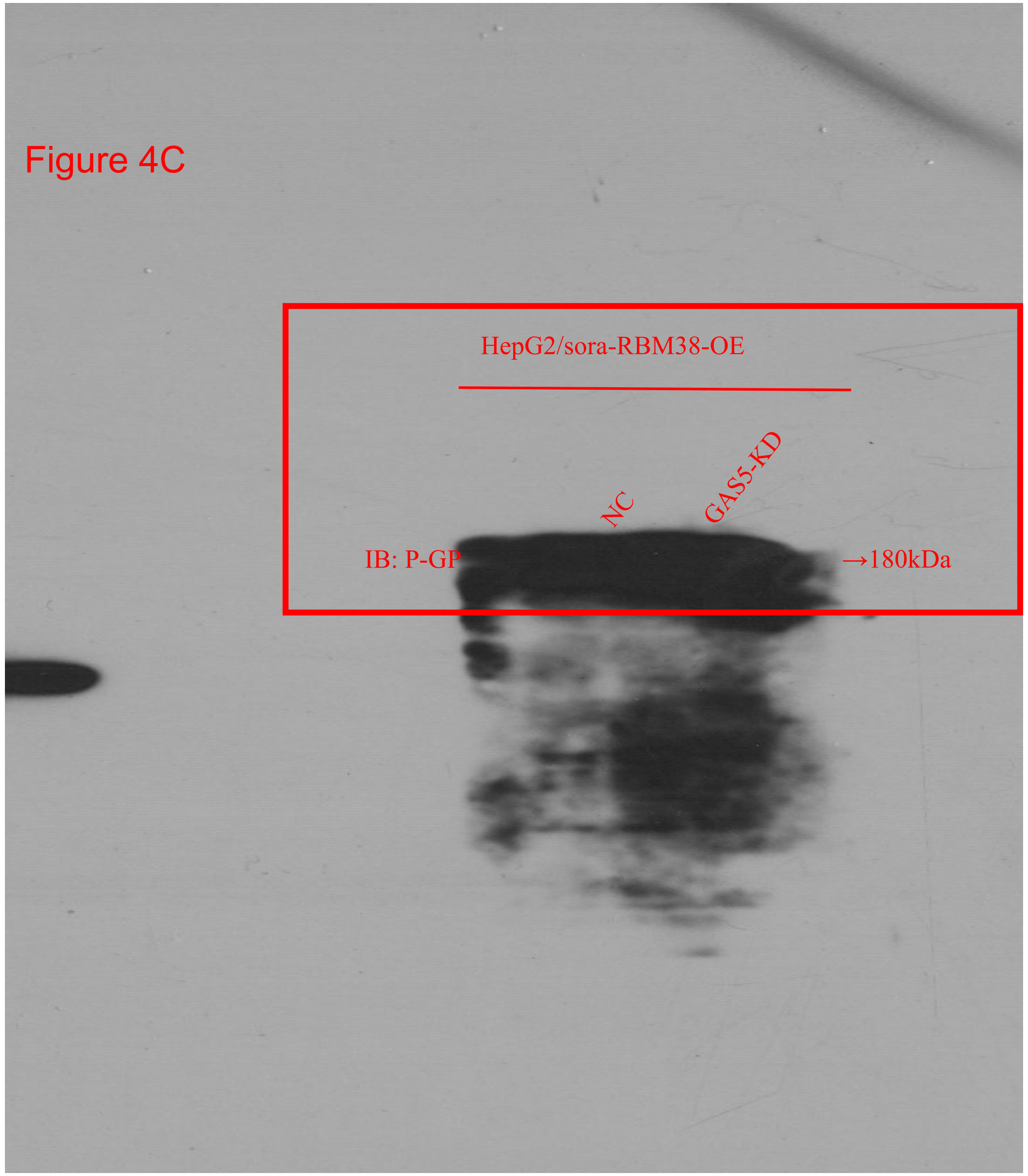

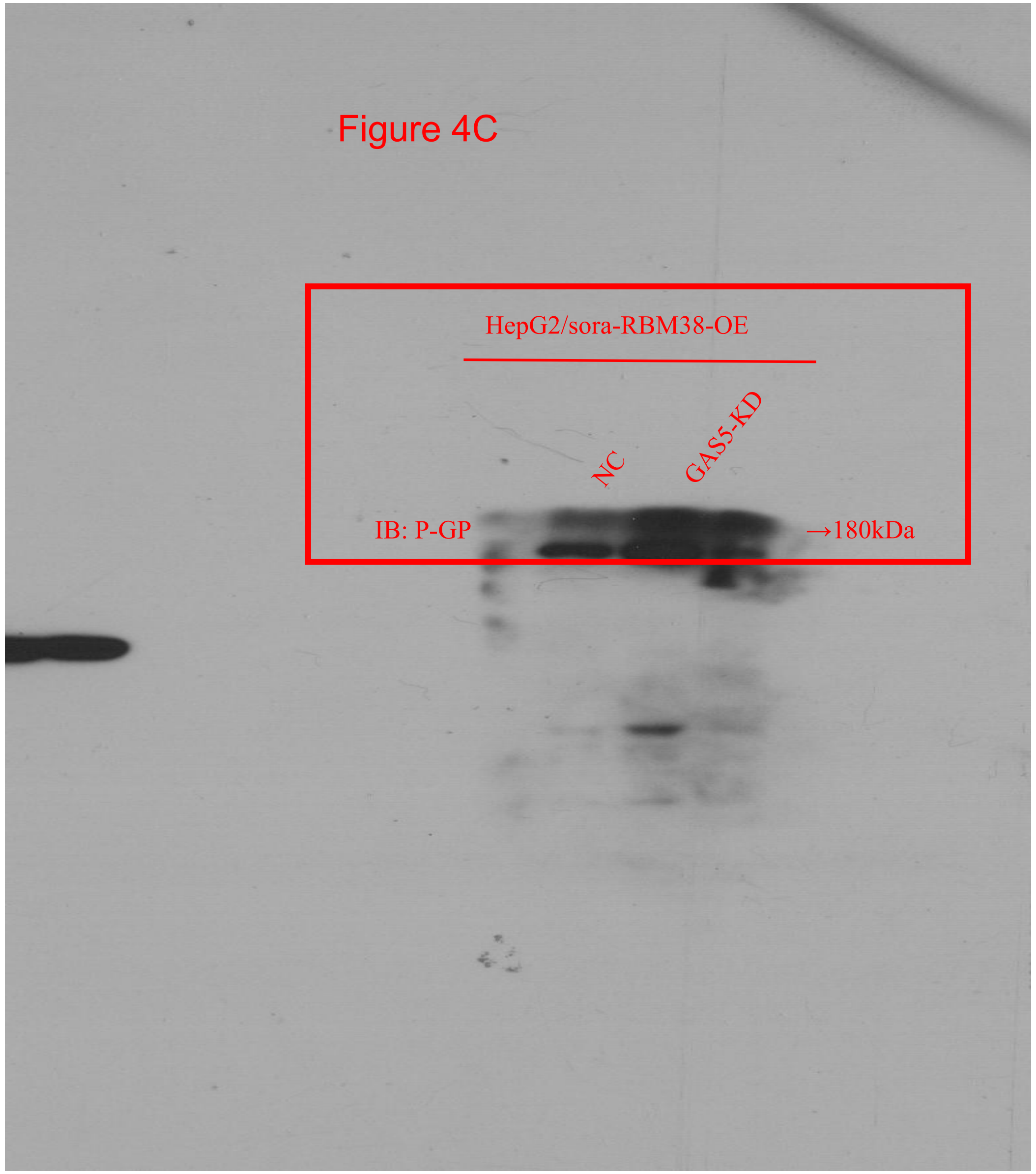

Figure 4C. Western blot analysis of P-GP, MRP1, and ABCG2 levels in 7721/sora-RBM38-OE+NC, 7721/sora-RBM38-OE+GAS5-KD, HepG2/sora-RBM38-OE+NC, and HepG2/sora-RBM38-OE+GAS5-KD cells.

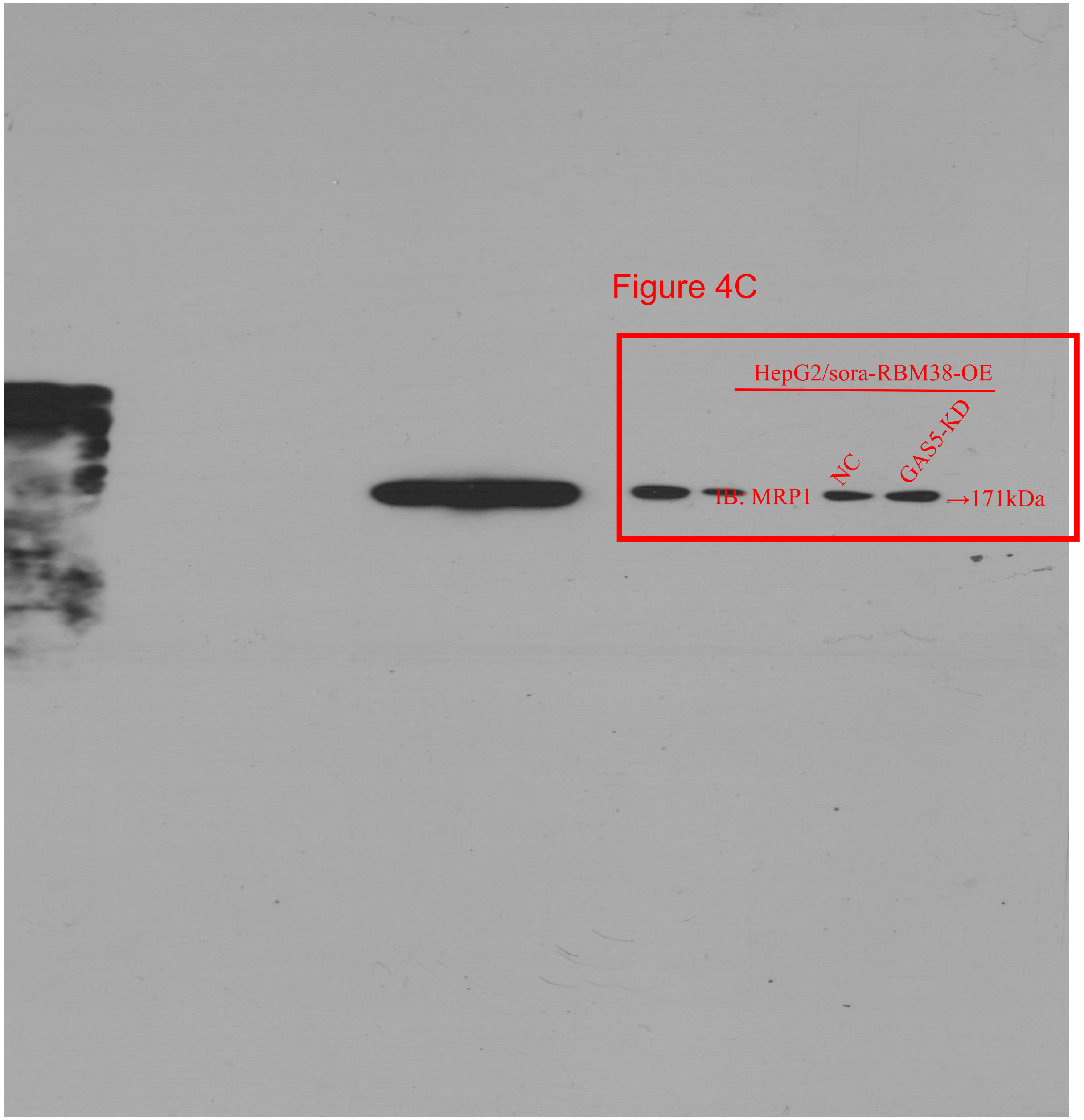

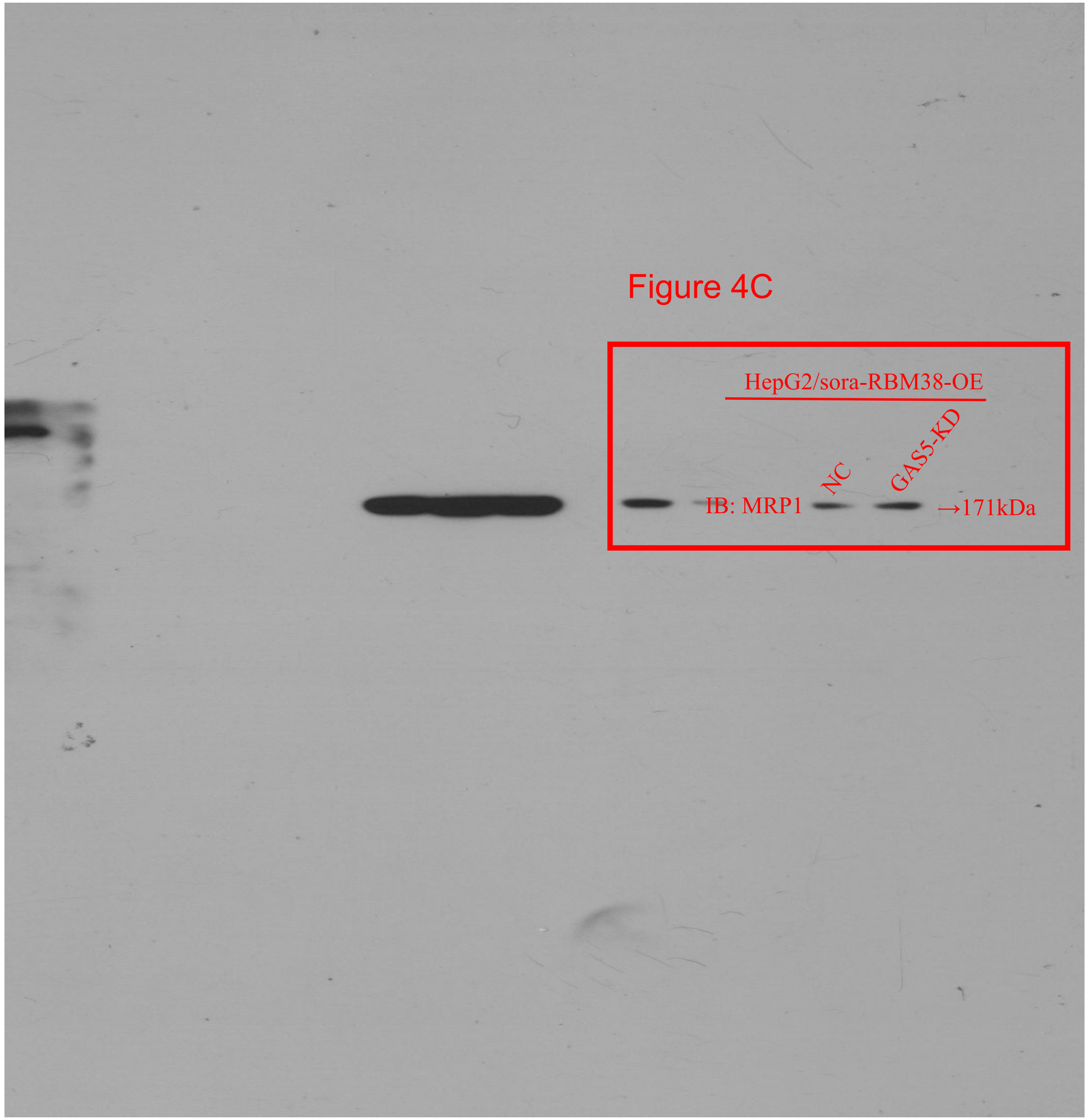

Figure 4C. Western blot analysis of P-GP, MRP1, and ABCG2 levels in 7721/sora-RBM38-OE+NC, 7721/sora-RBM38-OE+GAS5-KD, HepG2/sora-RBM38-OE+NC, and HepG2/sora-RBM38-OE+GAS5-KD cells.

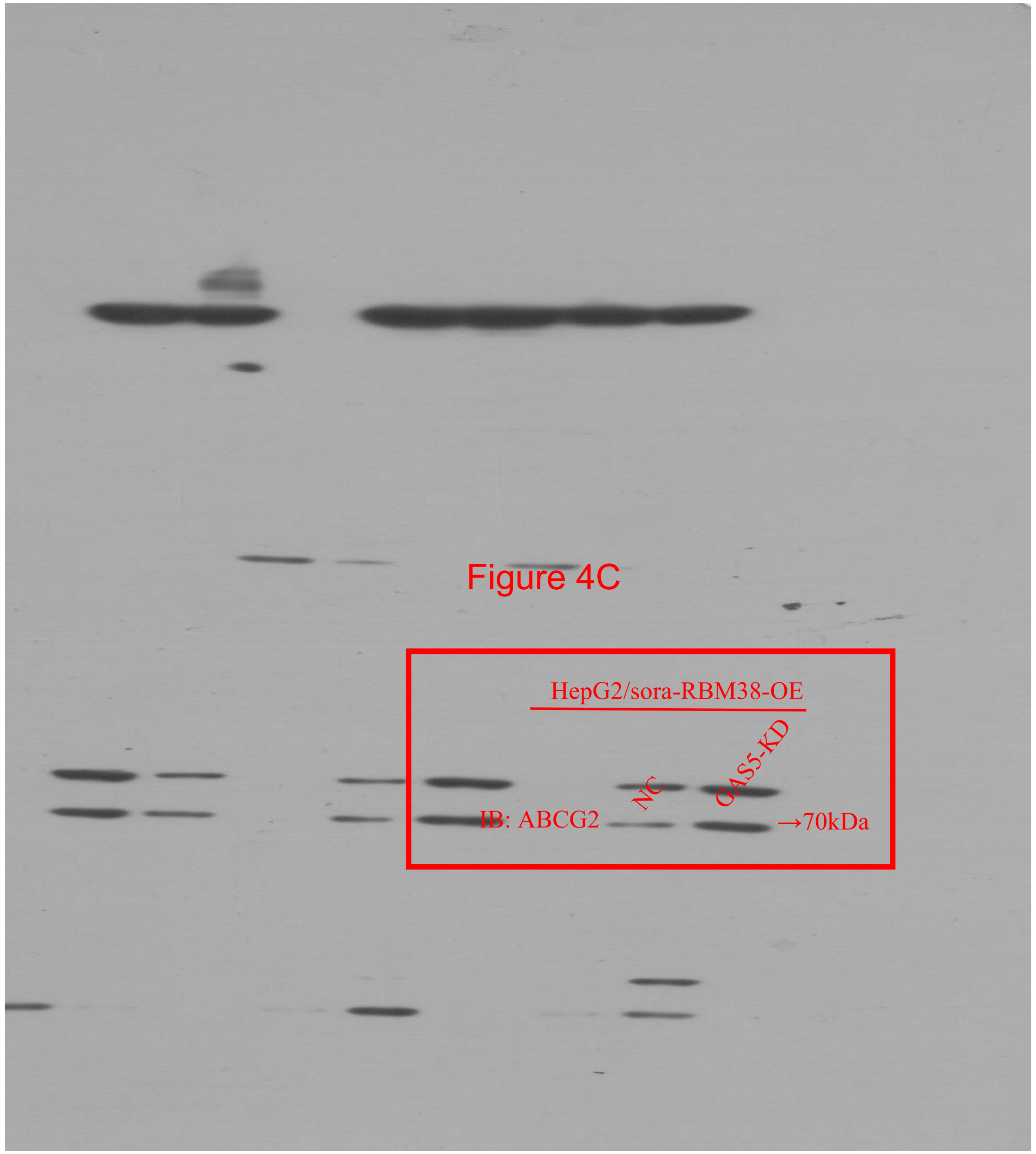

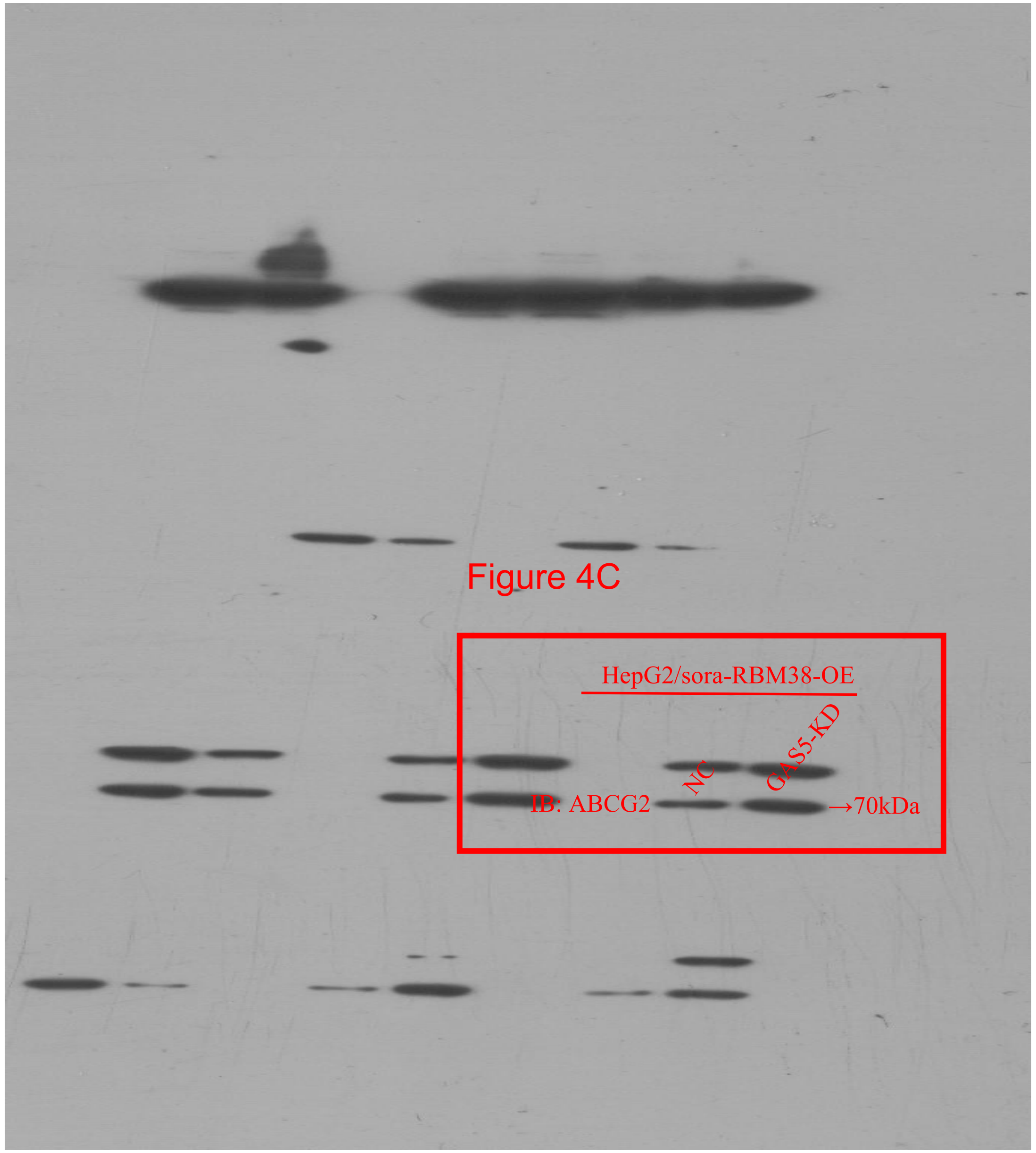

Figure 4C. Western blot analysis of P-GP, MRP1, and ABCG2 levels in 7721/sora-RBM38-OE+NC, 7721/sora-RBM38-OE+GAS5-KD, HepG2/sora-RBM38-OE+NC, and HepG2/sora-RBM38-OE+GAS5-KD cells.

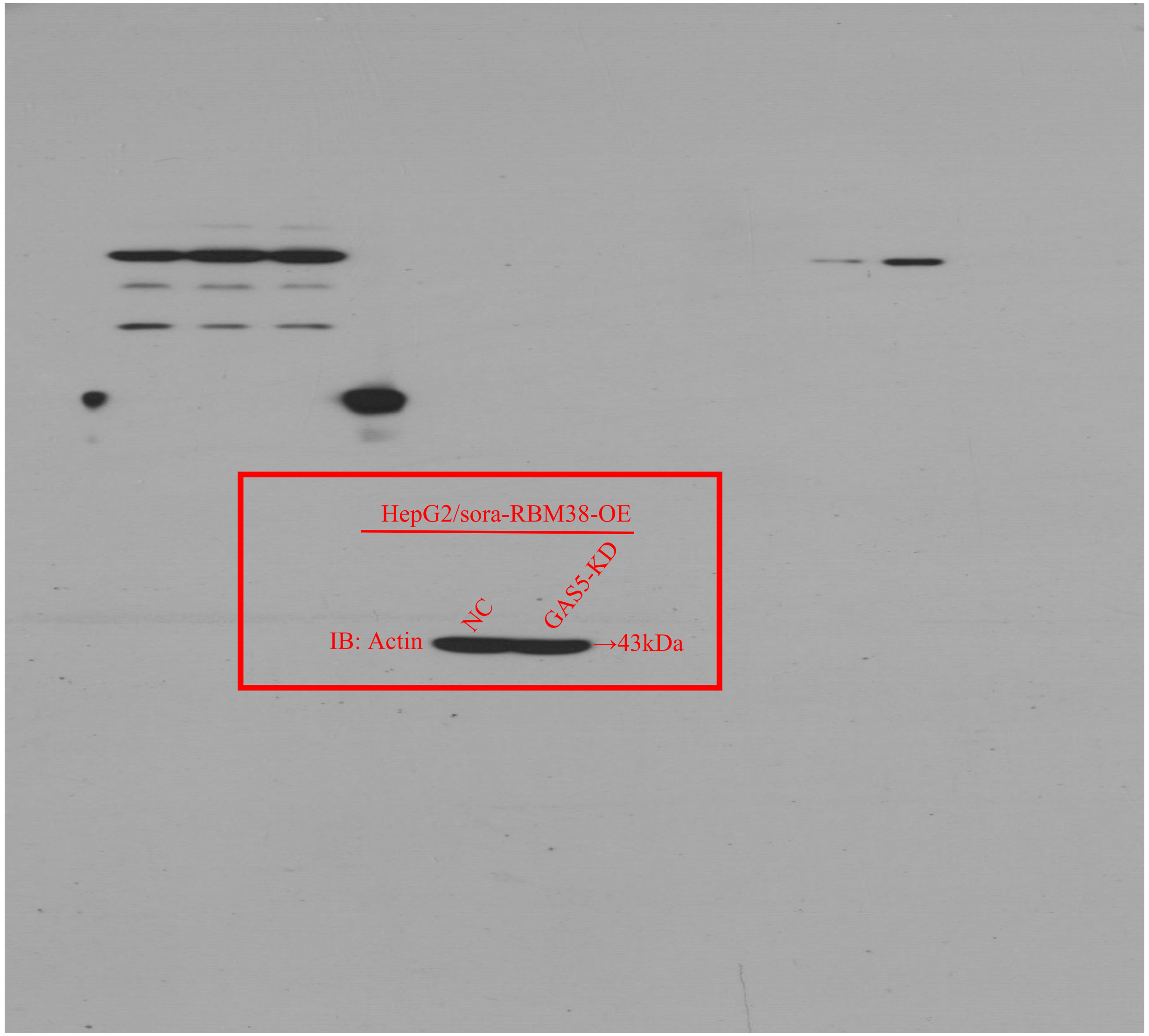

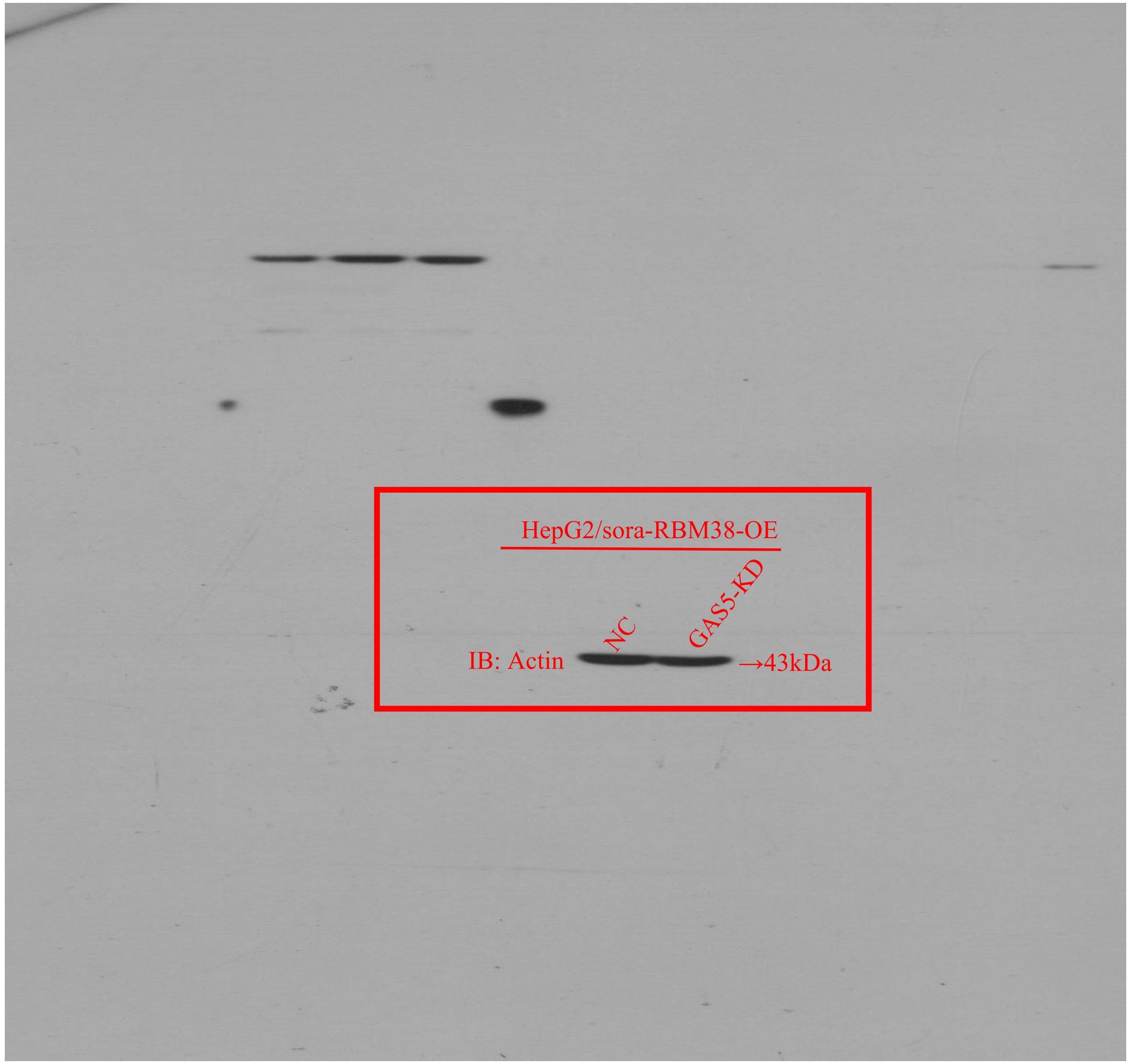

Supplement: Supplementary file 1 [file cancers-15-02897-s001.zip › File S1. The blot raw data.pdf]
