# Supplementary material for: RBM38 Reverses Sorafenib Resistance in Hepatocellular Carcinoma Cells by Combining and Promoting lncRNA-GAS5
Source: Cancers (Basel). 2023 May 24;15(11):2897. doi: 10.3390/cancers15112897 (PMC10252096; doi:10.3390/cancers15112897)
Supplement: Supplementary file 1 [file cancers-15-02897-s001.zip › File S2. The qPCR raw data.pdf]

Figure 1

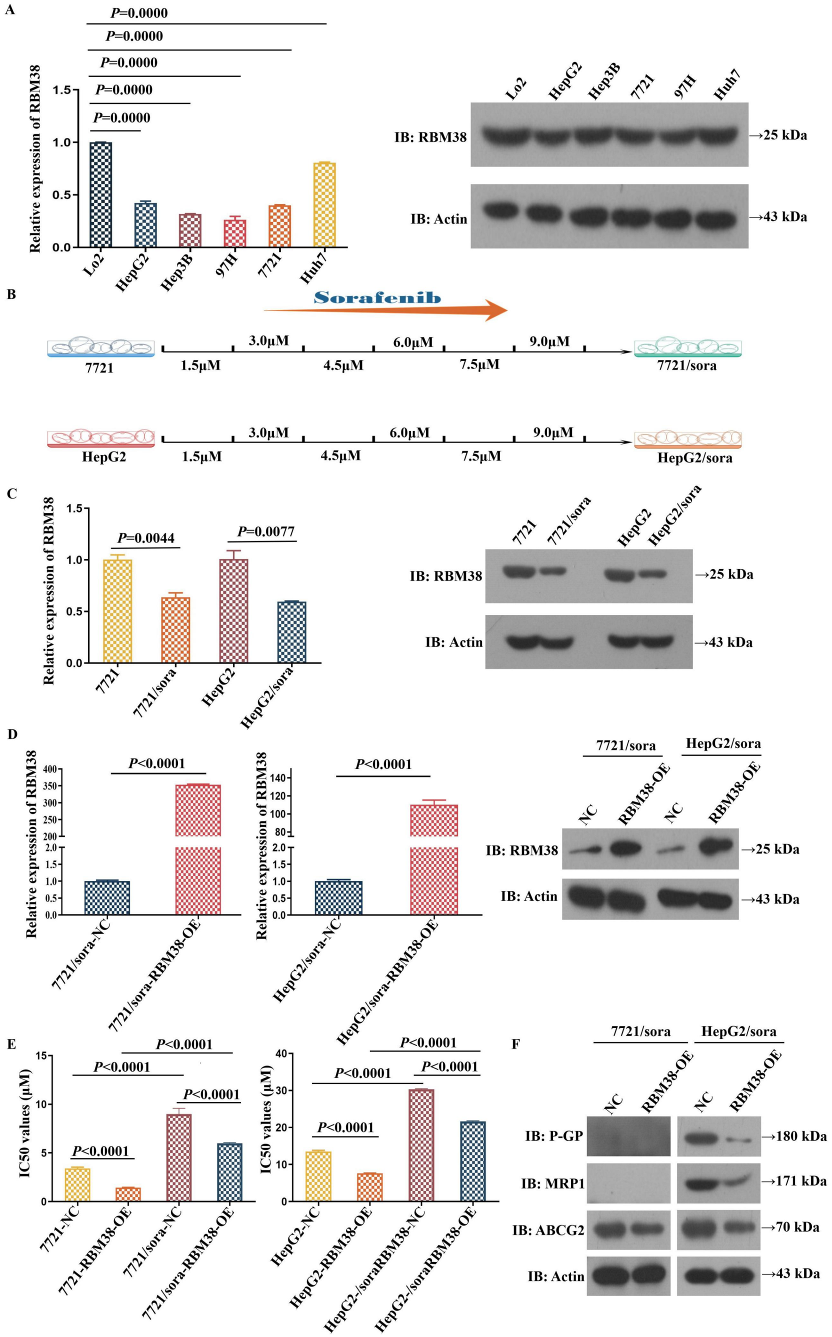
A
P=0.0000
P=0.0000
P=0.0000
P=0.0000
P=0.0000
Relative expression of RBM38
Lo2
HepG2
Hep3B
97H
7721
Huh7
IB: RBM38
IB: Actin
→25 kDa
→43 kDa
B
Sorafenib
3.0μM
6.0μM
9.0μM
1.5μM
4.5μM
7.5μM
7721
7721/sora
HepG2
HepG2/sora
C
P=0.0044
P=0.0077
7721/sora
HepG2/sora
D
P<0.0001
7721/sora-NC
7721/sora-RBM38-OE
HepG2/sora-NC
HepG2/sora-RBM38-OE
NC
RBM38-OE
E
IC50 values (μM)
7721-NC
7721-RBM38-OE
7721/sora-NC
7721/sora-RBM38-OE
HepG2-NC
HepG2-RBM38-OE
HepG2-/soraRBM38-NC
HepG2-/soraRBM38-OE
F
IB: P-GP
IB: MRP1
IB: ABCG2
→180 kDa
→171 kDa
→70 kDa

Figure1A. Real-time (RT)-PCR analysis of the expression and protein levels of RBM38 in HCC and normal liver cell lines.

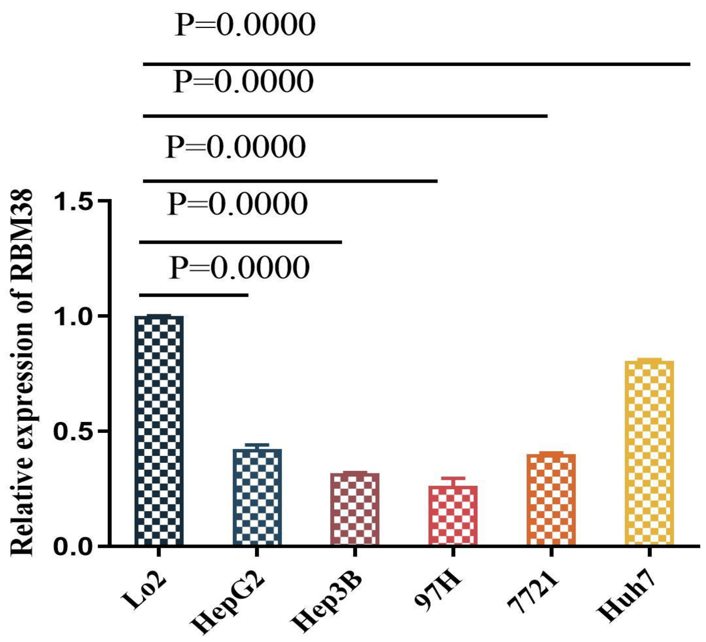

Figure1A. Real-time (RT)-PCR and analysis of the expression and protein levels of RBM38 in HCC and normal liver cell lines.

	Ct	Ct	mean Ct	detla Ct	mean detla Ct	detla detla Ct	mean detla detla Ct	2- detla detla Ct	mean 2- detla detla Ct		p-value
	RBM38	RPL13A	RPL13a	RBM38	RBM38	RBM38	RBM38	RBM38	RBM38	SD	
Lo2	21.03	14.04	14.1867	6.8433	6.8400	0.0033	0.0000	0.9977	1.0000	0.0040	
	21.03	14.42		6.8433		0.0033		0.9977			
	21.02	14.10		6.8333		-0.0067		1.0046			
HepG2	21.86	13.83	13.6433	8.2167	8.0867	1.3767	1.2467	0.3851	0.4223	0.0325	0.0000
	21.68	13.57		8.0367		1.1967		0.4363			
	21.65	13.53		8.0067		1.1667		0.4454			
Hep3B	22.37	14.00	13.8667	8.5033	8.4933	1.6633	1.6533	0.3157	0.3179	0.0038	0.0000
	22.34	13.64		8.4733		1.6333		0.3223			
	22.37	13.96		8.5033		1.6633		0.3157			
97H	22.75	14.36	14.0100	8.7400	8.7967	1.9000	1.9567	0.2679	0.2620	0.0576	0.0000
	23.16	13.85		9.1500		2.3100		0.2017			
	22.51	13.82		8.5000		1.6600		0.3164			
7721	24.32	16.08	16.1767	8.1433	8.1600	1.3033	1.3200	0.4052	0.4006	0.0080	0.0000
	24.32	16.38		8.1433		1.3033		0.4052			
	24.37	16.07		8.1933		1.3533		0.3914			
Huh7	22.53	14.03	14.1433	8.3867	8.3967	0.3000	0.3100	0.8123	0.8067	0.0097	0.0000
	22.53	14.03		8.3867		0.3000		0.8123			
	22.56	14.37		8.4167		0.3300		0.7955			

Figure1C. RT-PCR analysis of the expression and protein levels of RBM38 in PCL and drug-resistant HCC cell line.

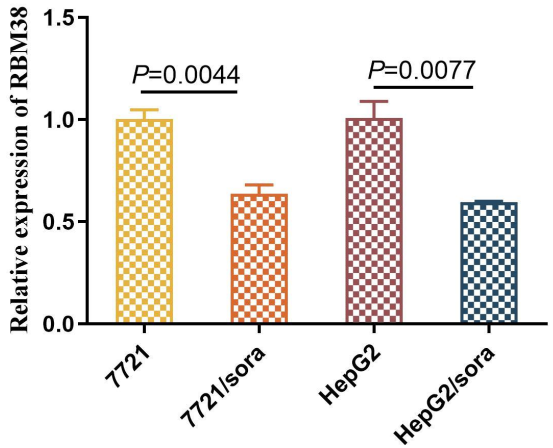

Figure1C. RT-PCR analysis of the expression and protein levels of RBM38 in PCL and drug-resistant HCC cell line.

	Ct	Ct	mean Ct	detla Ct	mean detla Ct	detla detla Ct	mean detla detla Ct	2- detla detla Ct	mean 2- detla detla Ct		p-value
	**RBM38**	**GAPDH**	**GAPDH**	**RBM38**	**RBM38**	**RBM38**	**RBM38**	**RBM38**	**RBM38**	**SD**	
	23.59	11.97		11.7133		0.0100		0.9931			
**7721**	23.69	11.88	11.8767	11.8133	11.7033	0.1100	0.0000	0.9266	1.0021	0.0805	
	23.46	11.78		11.5833		-0.1200		1.0867			
	25.00	12.74		12.2533		0.5500		0.6830			
**7721-sora**	25.01	12.72	12.7467	12.2633	12.3567	0.5600	0.6533	0.6783	0.6387	0.0727	0.0044
	25.30	12.78		12.5533		0.8500		0.5548			
	23.11	13.62		9.5233		-0.2167		1.1620			
**HepG2**	23.51	13.58	13.5867	9.9233	9.7400	0.1833	0.0000	0.8807	1.0066	0.1430	
	23.36	13.56		9.7733		0.0333		0.9772			
	23.67	13.28		10.4867		0.7467		0.5960			
**HepG2 -sora**	23.65	13.15	13.1833	10.4667	10.4867	0.7267	0.7467	0.6043	0.5960	0.0083	0.0077
	23.69	13.12		10.5067		0.7667		0.5878			

Figure1D. Overexpression (OE) efficiency of RBM38 in PCLs and drug-resistant HCC cells based on RT-PCR, compared with the negative control (NC).

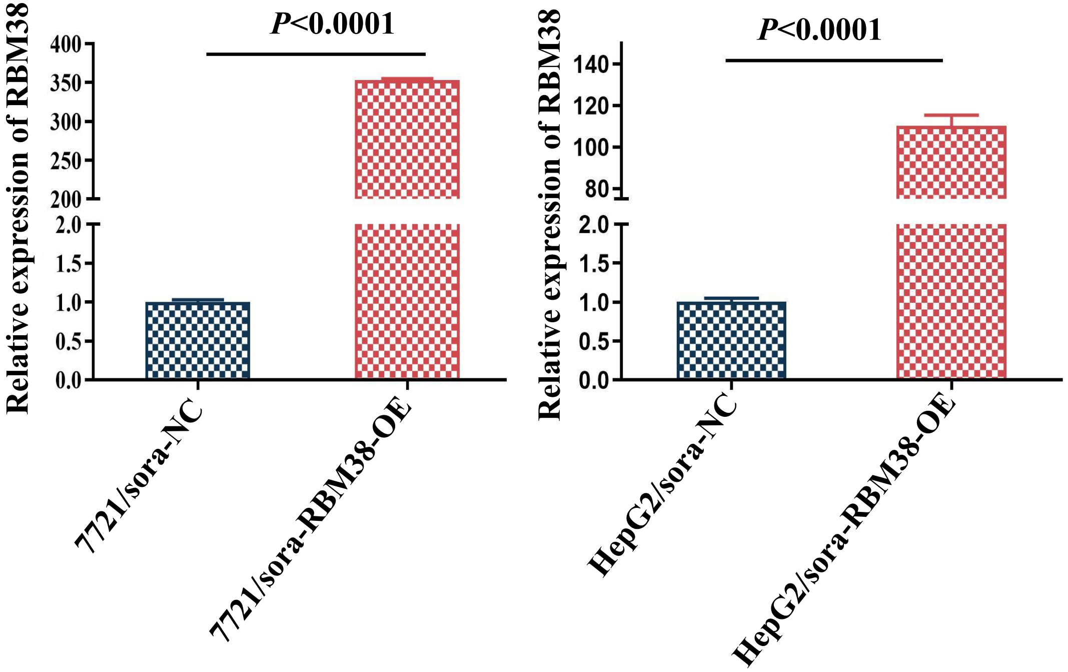

Figure1D. Overexpression (OE) efficiency of RBM38 in PCLs and drug-resistant HCC cells based on RT-PCR, compared with the negative control (NC).

7721/sora	Ct	Ct	mean Ct	detla Ct	mean detla Ct	detla detla Ct	mean detla detla Ct	2- detla detla Ct	mean 2- detla detla Ct		p-value
	RBM38	RPL13a	RBM38	RBM38	RBM38	RBM38	RBM38	RBM38	RBM38	SD	
NC	21.68	12.38	12.4300	9.2500	9.1667	0.0833	0.0000	0.9439	1.0009	0.0505	
	21.57	12.45		9.1400		-0.0267		1.0187			
	21.54	12.46		9.1100		-0.0567		1.0401			
RBM38-OE	13.27	12.76	12.5800	0.6900	0.7033	-8.4767	-8.4633	356.2304	352.9664	3.7310	0.0000
	13.28	12.47		0.7000		-8.4667		353.7697			
	13.30	12.51		0.7200		-8.4467		348.8992			

HepG2/sora	Ct	Ct	mean Ct	detla Ct	mean detla Ct	detla detla Ct	mean detla detla Ct	2- detla detla Ct	mean 2- detla detla Ct		p-value
	RBM38	RPL13a	RBM38	RBM38	RBM38	RBM38	RBM38	RBM38	RBM38	SD	
NC	21.66	13.62	13.6167	8.0433	8.0733	-0.0300	0.0000	1.0210	1.0022	0.0806	
	21.59	13.59		7.9733		-0.1000		1.0718			
	21.82	13.64		8.2033		0.1300		0.9138			
RBM38-OE	15.24	14.12	14.0733	1.1667	1.2933	-6.9067	-6.7800	119.9814	110.1383	9.0095	0.0000
	15.39	14.09		1.3167		-6.7567		108.1333			
	15.47	14.01		1.3967		-6.6767		102.3003			

Figure 3

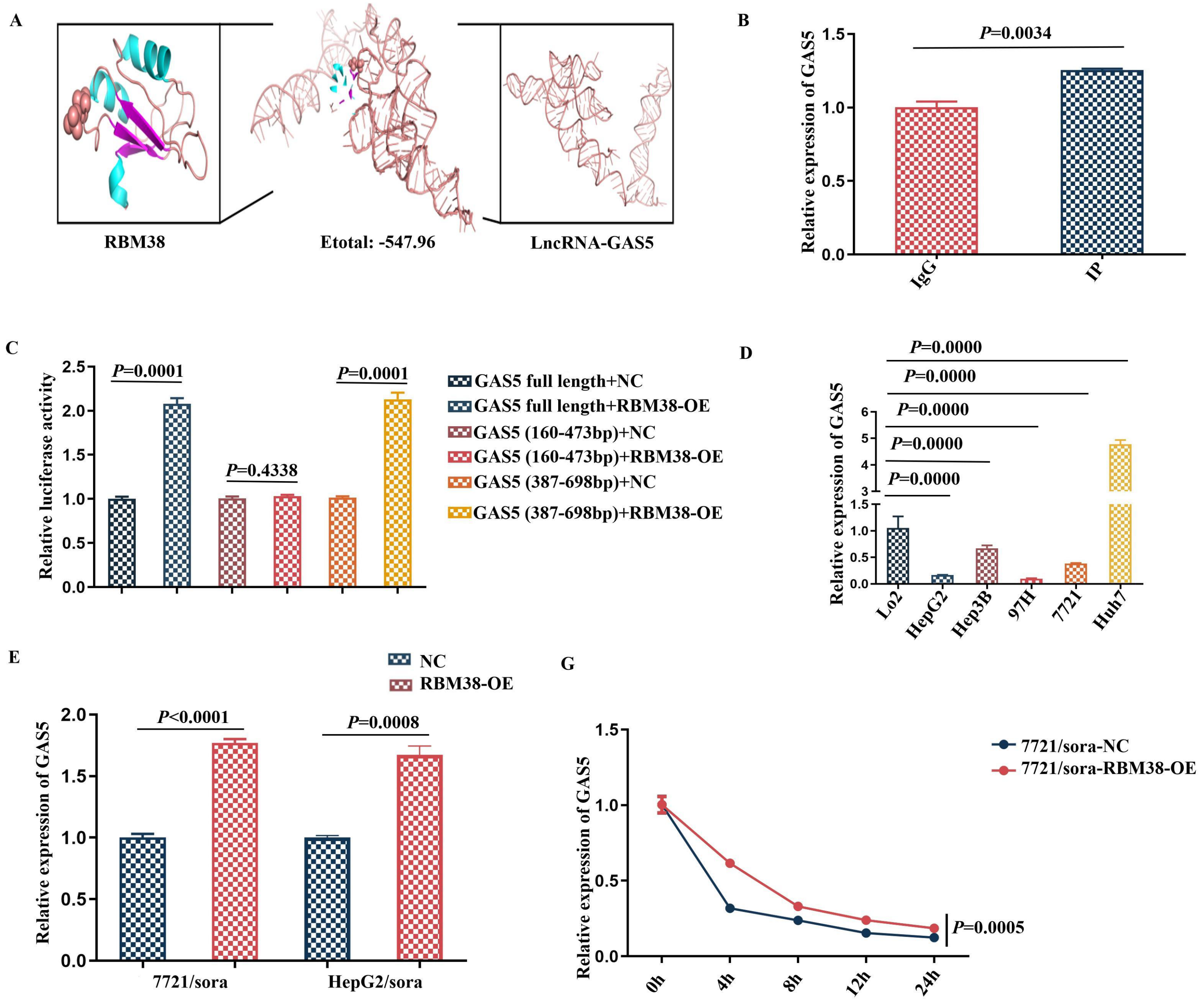
A
RBM38
Etotal: -547.96
LncRNA-GAS5
B
P=0.0034
Relative expression of GAS5
IgG
IP
C
Relative luciferase activity
P=0.0001
P=0.4338
P=0.0001
GAS5 full length+NC
GAS5 full length+RBM38-OE
GAS5 (160-473bp)+NC
GAS5 (160-473bp)+RBM38-OE
GAS5 (387-698bp)+NC
GAS5 (387-698bp)+RBM38-OE
D
P=0.0000
Relative expression of GAS5
Lo2
HepG2
Hep3B
97H
7721
Huh7
E
NC
RBM38-OE
P<0.0001
P=0.0008
Relative expression of GAS5
7721/sora
HepG2/sora
G
7721/sora-NC
7721/sora-RBM38-OE
Relative expression of GAS5
P=0.0005
0h
4h
8h
12h
24h

Figure3D. Real-time (RT)-PCR of lncRNA-GAS5 expression in HCC and normal liver cell lines.

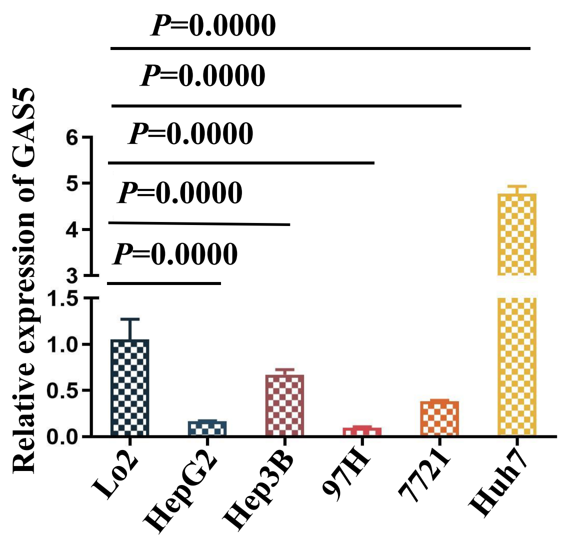

Figure3D. Real-time (RT)-PCR of lncRNA-GAS5 expression in HCC and normal liver cell lines.

	Ct	Ct	mean Ct	detla Ct	mean detla Ct	detla detla Ct	mean detla detla Ct	2- detla detla Ct	mean 2- detla detla Ct		p-value
	LncRNA-GAS5	RPL13A	RPL13a	LncRNA-GAS5	LncRNA-GAS5	LncRNA-GAS5	LncRNA-GAS5	LncRNA-GAS5	LncRNA-GAS5	SD	
**Lo2**	15.85	14.04	14.1867	1.6633	2.1000	-0.4367	0.0000	1.3535	1.0529	0.3791	
	16.96	14.42		2.7733		0.6733		0.6271			
	16.05	14.10		1.8633		-0.2367		1.1783			
**HepG2**	18.35	13.83	13.6433	4.7067	4.6767	2.6067	2.5767	0.1642	0.1677	0.0051	0.0155
	18.34	13.57		4.6967		2.5967		0.1653			
	18.27	13.53		4.6267		2.5267		0.1735			
**Hep3B**	16.76	14.00	13.8667	2.8933	2.6867	0.7933	0.5867	0.5770	0.6704	0.0949	0.1652
	16.55	13.64		2.6833		0.5833		0.6674			
	16.35	13.96		2.4833		0.3833		0.7667			
**97H**	19.61	14.36	14.0100	5.6000	5.4300	3.5000	3.3300	0.0884	0.0998	0.0099	0.0121
	19.35	13.85		5.3400		3.2400		0.1058			
	19.36	13.82		5.3500		3.2500		0.1051			
**7721**	19.75	16.08	16.1767	3.5733	3.4900	1.4733	1.3900	0.3601	0.3820	0.0212	0.0376
	19.66	16.38		3.4833		1.3833		0.3833			
	19.59	16.07		3.4133		1.3133		0.4024			
**Huh7**	16.64	14.03	14.1433	2.4967	2.4200	-2.1800	-2.2567	4.5315	4.7838	0.2668	0.0002
	16.57	14.03		2.4267		-2.2500		4.7568			
	16.48	14.37		2.3367		-2.3400		5.0630			

Figure3E. RNA level of lncRNA-GAS5 in RBM38-overexpressing cells detected by RT-PCR.

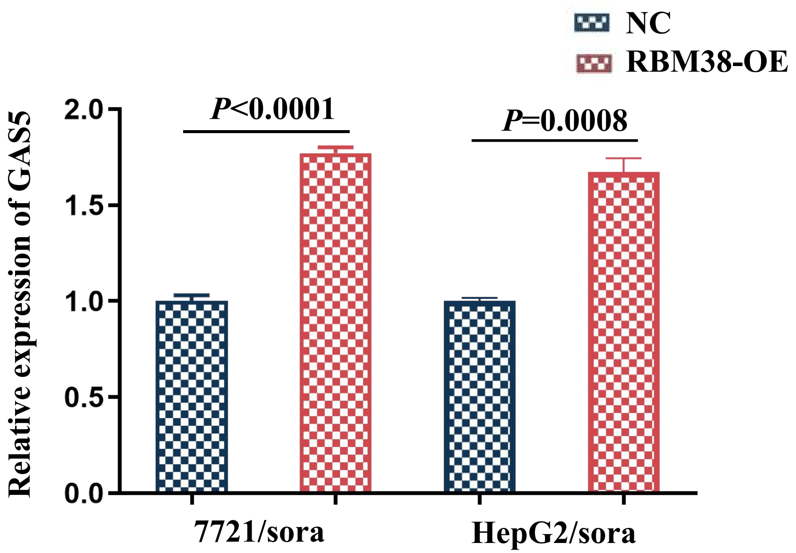

Figure3E. RNA level of lncRNA-GAS5 in RBM38-overexpressing cells detected by RT-PCR.

7721/sora	Ct	Ct	mean Ct	detla Ct	mean detla Ct	detla detla Ct	mean detla detla Ct	2- detla detla Ct	mean 2- detla detla Ct		p-value
	GAS5	RPL13a	GAS5	GAS5	GAS5	GAS5	GAS5	GAS5	GAS5	SD	
NC	14.29	12.38	12.4300	1.8600	1.9333	-0.0733	0.0000	1.0521	1.0009	0.0520	
	14.36	12.45		1.9300		-0.0033		1.0023			
	14.44	12.46		2.0100		0.0767		0.9482			
RBM38-OE	13.67	12.76	12.5800	1.0900	1.1100	-0.8433	-0.8233	1.7942	1.7700	0.0530	0.0001
	13.66	12.47		1.0800		-0.8533		1.8067			
	13.74	12.51		1.1600		-0.7733		1.7092			

HepG2/sora	Ct	Ct	mean Ct	detla Ct	mean detla Ct	detla detla Ct	mean detla detla Ct	2- detla detla Ct	mean 2- detla detla Ct		p-value
	GAS5	RPL13a	GAS5	GAS5	GAS5	GAS5	GAS5	GAS5	GAS5	SD	
NC	18.35	13.62	13.6167	4.7333	4.7033	0.0300	0.0000	0.9794	1.0003	0.0305	
	18.34	13.59		4.7233		0.0200		0.9862			
	18.27	13.64		4.6533		-0.0500		1.0353			
RBM38-OE	18.12	14.12	14.0733	4.0467	3.9633	-0.6567	-0.7400	1.5764	1.6731	0.1225	0.0008
	18.07	14.09		3.9967		-0.7067		1.6320			
	17.92	14.01		3.8467		-0.8567		1.8108			
